# Emulation of the subjective experience of visual dorsal stream dysfunction: a description of three in depth case studies

**DOI:** 10.3389/fnhum.2024.1496811

**Published:** 2025-01-06

**Authors:** Helen St Clair Tracy, Nicola McDowell, Gordon N. Dutton, John Ravenscroft, Isobel Hay, Andrew Blaikie

**Affiliations:** ^1^Infection and Global Health Division, School of Medicine, University of St Andrews, St Andrews, United Kingdom; ^2^Institute of Education, Massey University, Auckland, New Zealand; ^3^Glasgow Caledonian University, Glasgow, United Kingdom; ^4^Moray House School of Education and Sport, The Scottish Sensory Centre, University of Edinburgh, Edinburgh, United Kingdom; ^5^NHS Dumfries and Galloway, Dumfries and Galloway, United Kingdom

**Keywords:** cerebral visual impairment, CVI, simultanagnosia, simultanagnostic vision, dorsal stream dysfunction

## Abstract

These case studies explore the subjective visual experiences of individuals with cerebral visual impairment (CVI), specifically dorsal stream dysfunction (DSD) characterized by simultanagnosia. Through three in-depth case studies, this work documents the challenges these individuals face when navigating cluttered environments. The individuals were asked to describe their visual experiences while watching videos of varying complexity, with the future aim of creating a simulation of simultanagnosia. This process revealed a dynamic constriction of their attentional visual fields as scene complexity increased, and vice versa. Notably, the volunteers experienced a phenomenon where their vision could “get stuck” on specific items, with an apparent concurrent reduction in their ability to perceive and describe visual information as visual clutter increased. These consistent observations indicate that the symptoms of simultanagnosia are not simply limited to perceiving one or two objects at a time but can vary dynamically in response to environmental complexity. They enhance our understanding of how DSD impacts visual search and perceptual experiences, prompting us to propose the term “simultanagnostic vision” to describe this more nuanced and dynamic manifestation of CVI. The results are critical for developing effective interventions and optimizing support strategies for individuals affected by DSD, particularly children at sensitive developmental stages. Furthermore, we recommend deeper investigation into how different visual processing streams in the brain interconnect and influence each other, which may open new avenues for targeted therapeutic interventions.

## 1 Introduction

Our vision is constructed through the dynamic interplay of multiple interconnected brain regions that process visual information from our surroundings, engaging over 40% of the brain’s capacity (Hubel, 1995). Although specific regions are associated with particular aspects of visual processing, these regions operate in highly interconnected networks (Bennett et al., 2021). Consequently, damage to or dysfunction within the brain can frequently impair vision, leading to cerebral visual impairment (CVI). A widely used definition of CVI describes it as a ‘verifiable visual dysfunction which cannot be attributed to disorders of the anterior visual pathways or any co-occurring ocular impairment’ (Sakki et al., 2018). However, this definition offers limited insight into the subjective visual experiences of individuals affected by CVI. According to the ICD-11 classification, CVI is categorized under “Specific visual dysfunctions” (9D92), which “refer to functional deficits in higher cerebral centers. Such dysfunctions may exist with or without visual impairment of the eyes and the lower visual system” (World Health Organization, 2024). The brain’s image processing is highly complex, with different regions responsible for specific visual functions, making the outcomes of brain injuries or dysfunctions highly varied and likely unique to each individual. This poses a critical challenge: how do we come to understand how affected individuals perceive their worlds, and how can we use this information to provide effective support as a sequel to diagnosis?

Vision serves four primary functions: guiding movement, enabling social interaction, facilitating access to information, and promoting learning. This understanding is particularly important when teaching children (Hyvärinen and Jacob, 2011). For those with CVI, barriers to learning include low visual acuity, reduced contrast sensitivity, restricted visual fields, impaired movement perception (Pamir et al., 2021) and limited perceptual capacity, especially in terms of parallel processing, related to reduced nerve fiber numbers in the dorsal visual stream pathways (Bennett et al., 2019).

The dorsal stream processes visual data from the occipital and middle temporal lobes, relaying it to the posterior parietal lobes and the interlinked subcortical pathways to enable the visual guidance of movement (Glickstein et al., 2011). This process is typically non-conscious, allowing actions such as running upstairs without active thought (Milner and Goodale, 2006; Goodale and Milner, 2013). In contrast, the ventral stream relays visual information from the occipital lobes to the temporal lobes, facilitating recognition by matching incoming image data with stored memories (Milner and Goodale, 2006; Goodale and Milner, 2013). Both streams are processed in higher cerebral centers, and dysfunctions in either can lead to “perceptual visual dysfunction” (Pehere and Dutton, 2021). However, these functions are not stand-alone processes. The vertical occipital fasciculi (VOF), comprise key white matter pathways, linking the bilateral dorsal (underpinning visual guidance of movement and spatial attention) and ventral (recognition) visual streams, thought to play a key role in integration of these distinct visual functions, in those with typical vision (Yeatman et al., 2014). The connectivity of both VOFs enables integration of visual information, supporting coherent perception and visual memory. Disruptions in the VOF can impair these functions, emphasizing the importance of viewing visual processing as an interconnected network rather than isolated pathways (Bauer and Merabet, 2024). How these interconnected streams are affected when one of the visual centers is not processing typically is not well understood and may vary uniquely among individuals. Therefore, the complexity and individuality of such impairments make it essential to fully ascertain each person’s CVI etiology and pattern of visual affectation to provide optimal support.

Dorsal stream dysfunction (DSD), often seen in those with CVI, can result in multiple visual impairments, such as optic ataxia, where individuals struggle with using vision to accurately guide their movements, rendering everyday activities like writing and eating difficult (Vighetto and Salmon, 2007). DSD can also interfere with finding objects in cluttered environments, leading to slower and less precise visual search (Bennett et al., 2020; Zhang et al., 2022). Balint syndrome is the most severe variant of this condition (Gillen and Dutton, 2003). Unilateral parietal lobe pathology can also cause hemi-inattention, where individuals become less aware of one side of their visual field, particularly when the right parietal lobe pathology leads to left sided inattention, with severe cases resulting in visual neglect (Vighetto and Salmon, 2007). While CVI-related impairments in visual acuity, contrast sensitivity, and visual field are well understood, DSD and its effects remain less well recognized, despite detailed reports of simultanagnosia in adults (Michel and Henaff, 2004). Simultanagnosia, an element of DSD, manifests as difficulty perceiving more than one or two objects in a visual scene at once (Hokken et al., 2024), and in severe cases, an inability to simultaneously perceive the parts of a single object (Coslett and Lie, 2008). Simultanagnosia is typically described by the challenges it causes, but questions remain about its underlying nature and how it affects vision at a subjective level. Understanding this is crucial for providing adequate support.

To better understand simultanagnosia, we collaborated with three adults with formally diagnosed DSD to ascertain the nature of their visual experiences and from this, to create a video simulation. By in-depth analysis of what they described seeing during real time observation of photos and videos and discussing their responses with them, we aimed to visually replicate their descriptions, with the aim of helping others affected by this condition to better understand the nature of their vision. This paper presents their accounts, how they contributed to our understanding of simultanagnosia, for the development of a simulation to depict their experiences. We also discuss public responses to the simulation, which was made freely available online. The following narrative outlines the process that led to new insights into simultanagnosia, opening avenues for future research, potential diagnostic methods, and even potential therapeutic options. Additionally, the project prompted revisions in how CVI can best be explained, particularly the importance of distinguishing between the ventral and dorsal pathways in CVI and the need to understand how other pathways and regions in the brain are affected and react when visual areas are not processing typically. The events are presented in chronological order to reflect the unfolding nature of this inquiry.

## 2 Background

In 2016, although it was generally assumed that clutter and complexity negatively impacted children with CVI, there was limited published evidence, with only one small case study available (Little and Dutton, 2015). An email discussion between a CVI expert, an adult with acquired CVI, and the mother of a child with CVI explored the nature of CVI, particularly simultanagnosia, from their unique perspectives. It emerged that the adult with CVI found environments with less clutter not only easier to navigate but also conducive to better visual perception. This observation had not been previously described and prompted our group to informally test this concept framework.

To investigate further, we initially asked the adult with acquired CVI to view photographs and later expanded the inquiry to include two additional adults with CVI, including DSD, viewing videos. The three participants, anonymized as Cases 1–3, have all been the subject of previously published findings.

### 2.1 Case 1 medical history

As a 16-year-old, Case 1 initially presented with an acute intracerebral hemorrhage caused by the rupture of a left occipital arteriovenous malformation (AVM). Emergency neurosurgical intervention was performed, resulting in the removal of her left occipital lobe. Post-operatively, Case 1 developed right homonymous hemianopia due to the damage sustained by her occipital lobe. In addition, she experienced a transient episode of left-sided hemiparesis, which resolved shortly thereafter. Following recovery from surgery, Case 1 experienced persistent challenges with visual perception. She struggled to cope in crowded environments and had significant difficulties processing complex visual scenes. These symptoms are consistent with the dysfunction of visual processing pathways associated with the left occipital lobe damage. This published case (McDowell and Dutton, 2019), highlights the long-term visual and cognitive sequelae resulting from the hemorrhagic event and subsequent surgical intervention.

### 2.2 Case 2 medical history

Case 2 was diagnosed with sagittal suture stenosis and scaphocephaly at age 3 months. A follow-up at a tertiary center at age 3, including a brain MRI, found normal development. However, Case 2’s mother recalls that as a pre-schooler, he “…could not remember or retain information about shapes.” At age 5, pediatric examination identified severe generalized hypotonia with normal muscle power and noted issues with social communication with peers. Neurological and genetic investigations, including MRI of the brain and spine, as well as peripheral nerve conduction tests, revealed no cause for hypotonia. At age 11, Asperger’s syndrome (ICD-10 criteria) was diagnosed after multidisciplinary assessment. An initial ocular examination at age 12 was within normal limits (unaided VAs of 6/9 OD and 6/24 OS, corrected 6/6 OD and 6/9 OS) with automated visual field assessments being normal. Mild left optic nerve pallor was noted. At age 13, tertiary neurological assessment identified Balínt syndrome, and recommendations were made for support as a child with severe visual perceptuomotor disability. At 14 years of age, Case 2 developed severe anxiety and depression, which required medication. At age 15, follow-up ocular examination found stable visual acuities although constricted visual fields were noted on perimetry. No retinal signs were noted. On review at age 17 the clinical findings were stable. Appendix 2: Poster: “Visual and Motor Deterioration in Asperger’s Syndrome,” is a case report on Case 2. Case 2 has featured in previous published research further documenting assessment tests (Hay et al., 2016).

### 2.3 Case 3 medical history

Case 3 was born at 38 weeks’ gestation with a normal birth weight, but exhibited floppiness at birth, which persisted for several days. She also manifested unexplained cyanotic episodes within the first 72 h of life. Her motor development was delayed; she sat with support at 8 months, crawled at 1 year, and walked independently at 14 months. Throughout early childhood, she experienced frequent trips and falls. As a young child she developed a habit of tugging on her parents’ clothes while walking, a behavior that persisted into teenage years, indicative of lower visual field impairment. During infancy, she often used her left hand while her right arm remained at her side. Toys were placed in her right hand to encourage its use. By 8–9 months of age, she could finger-feed independently, but by her late teens, she continued to struggle with using cutlery, tying shoelaces, and folding clothes. In primary school, Case 3’s handwriting difficulties and motor clumsiness were noted. A Test of Visual Perceptual Skills assessment revealed significant challenges with visual discrimination and closure. It was also apparent that she recognized shapes by tracing their outlines with a finger rather than relying on vision alone. Pediatric examination revealed general hypotonia and ligamentous laxity, though no significant neurological impairment was found. At the age of 7 she was referred for multi-disciplinary assessment for possible social-communication disorder due to difficulties with peer relationships and a dependence on routines. A diagnosis of autism spectrum disorder (ASD) was made. By the age of nine, her behavior was indicative of severe visual field restriction. She often tripped over low-lying objects, bumped into doorframes, and knocked food off her plate. She also had difficulty with facial recognition. Despite these symptoms, ocular examination was normal, but perimetric examination confirmed severe visual field constriction, subtending only five degrees. However, MRI scans of the brain and anterior visual pathways were normal. At 13 and 14 years old, serial hand movement examinations using slow-motion video identified bilateral optic ataxia, which was most pronounced in the left (dominant) hand and left hemifield. This was consistent with bilateral, or predominantly right parieto-occipital dysfunction; Balínt syndrome was subsequently confirmed by tertiary neurological opinion. Case 3 has featured in previous published research further documenting assessment tests (Hay et al., 2020).

## 3 Methodology

To examine the potential impact of crowding and clutter on visual perception, photographs were initially taken in various settings with differing levels of complexity. This was followed by the creation of videos, beginning in a small shop that was closed for filming. Visual attention is mapped across the entire body, not just the eyes and head, and is continuously re-mapped as individuals move through their surroundings. This process is essential for avoiding collisions and ensuring accurate visual guidance for reaching (Reed et al., 2007). Replicating this body-centric perspective in videos required the development of a novel method that, to our knowledge, had not been previously attempted. Our initial approach used two synchronized GoPro cameras mounted in a 3D-printed harness; however, this setup produced footage that was unstable and visually disorienting.

A simpler and more effective technique was developed by holding a digital camera at navel height with both hands, keeping the arms close to the body for stability. This method allowed the camera to move naturally with the individual, achieving the desired body-centered perspective. Using this approach, additional videos were recorded in both cluttered and uncluttered environments. From several hours of footage, six short clips were selected to represent a range of settings with varying levels of complexity and movement, enabling an assessment of the impact of these factors on visual perception.

This study was conducted as a qualitative exploration rather than adhering to a formal research protocol; accordingly, the methodology was not standardized. Instead, the design of the sessions described below was guided by the team’s expertise. The methods applied evolved organically, adapting in response to feedback from the volunteer participants. This flexible approach is exemplified in the findings from the following three case studies.

## 4 Data

### 4.1 Case 1

Case 1 is an academically high-achieving professional woman, who is open, and good-humored, with a love of sports and outdoor activities. She acquired CVI while at school, which led to considerable difficulties that required resilience and determination to overcome.

As an adult with acquired simultanagnosia, Case 1 underwent diagnosis and engaged in a self-developed program of “conscious vision strategies” (Appendix 1). These exercises were designed to transform her unconscious visual processing into a more conscious experience. By gradually honing this approach, she developed the ability to better recognize and understand the nature of her visual perceptions. This process revealed that her visual experience varied dynamically, with the extent of what she could perceive fluctuating, in relation to the visual complexity of her surroundings.

Case 1 gradually realized that as the visual complexity of her environment increased, her visual field became more restricted. In 2016, to evaluate this phenomenon and create a simulation for the launch of a CVI website, Case 1 was provided with photos of varying complexity, including images from inside shops and open countryside. She was asked to describe what she could see (“say what you see”) in each image. Her qualitative responses confirmed that her vision varied with the complexity of the scene: the more complex the visual environment, the fewer details she was able to perceive and describe.

**Figure 1:**
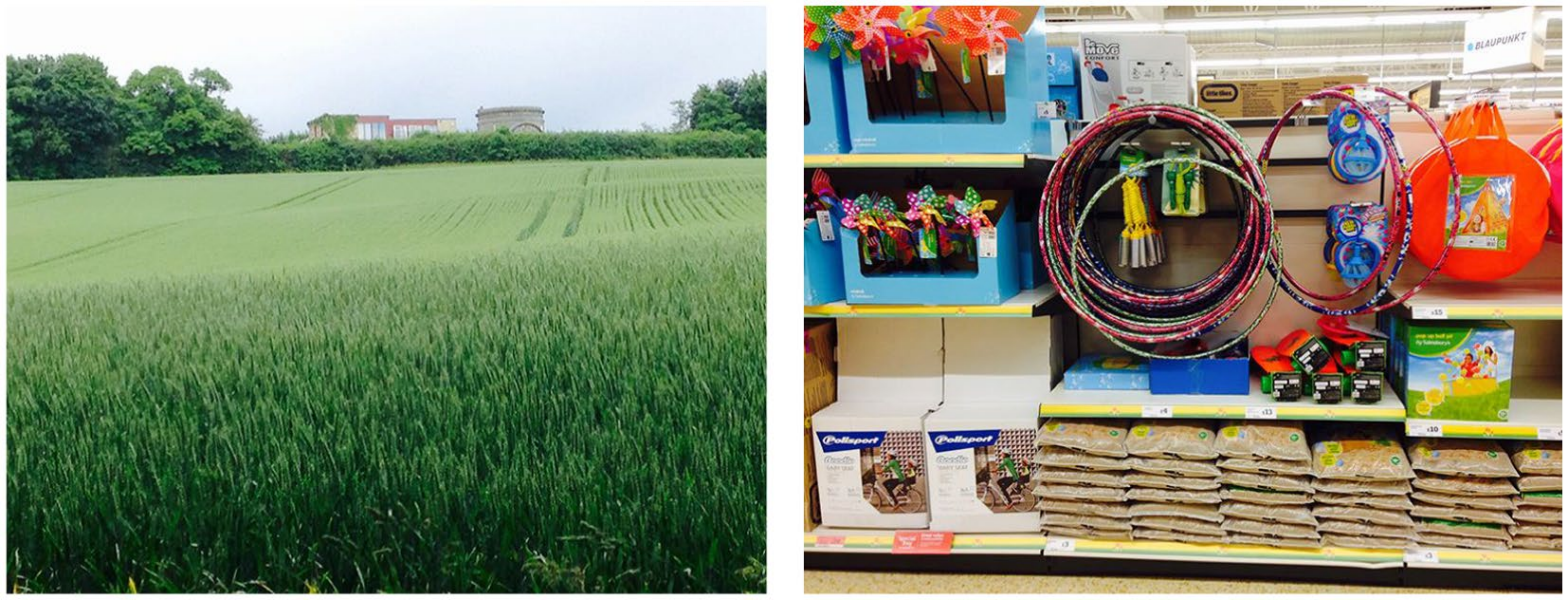
Two of the photographs used by CVI Scotland when attempting to create a simulation of simultanagnosia.

**Figure 2:**
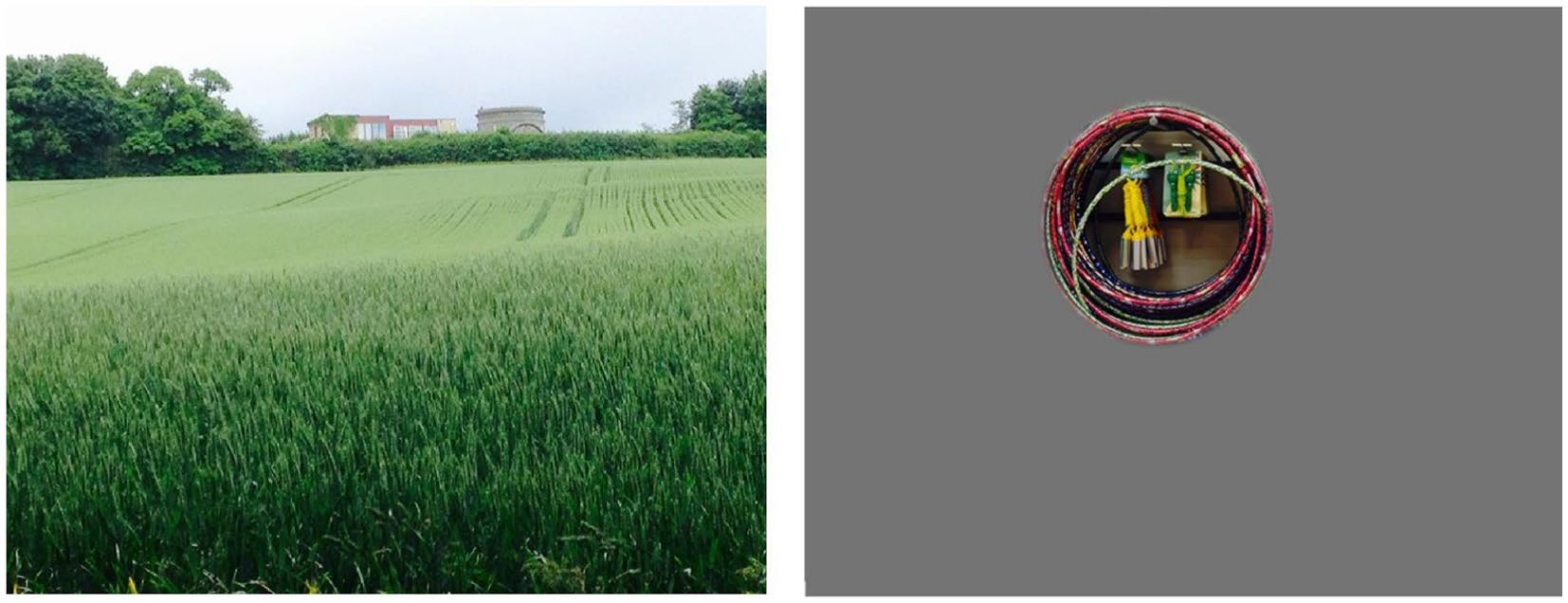
The application of a filter to cover areas that were not perceived, demonstrating an initial attempt at simulating simultanagnosia. Note that while the content inside the hoops was also not perceived, at that stage the significance of this was not yet recognized, and thus it was not initially covered.

Case 1, when viewing the image of the field on the left (see Figure 1), remarked that she could “*study the buildings at the back of the image and they become clearer*” and felt that she had seen “*every element in the photo: grass, hedges, trees, buildings, and sky.*” In contrast, when looking at the photo on the right, Case 1 noted, “*I was straight away drawn to the round hoops in the middle. If I tried to move my gaze away, it quickly got drawn back to them. A bit like a magnet, I guess*.” Her responses suggest that while Case 1 could perceive the entire scene in the simpler image, her attention was involuntarily fixed on specific elements in the more complex image, limiting her overall visual awareness. Full responses to these and other photos are available on the University of St Andrews School of Medicine website (University of St Andrews, 2024). What was not seen by Case 1 in the images was covered with a fog filter (Figure 2).

Reviewing the two images and the impact of simultanagnosia on vision, it became evident that visual clutter significantly impaired Case 1’s ability to perceive the scene. In 2018, Case 1 expressed a desire to further explore her perception of moving images in videos and help others with CVI better understand their vision. Before creating the videos, Case 1 walked through a busy gift shop, using a video camera to capture its surroundings, while explaining the challenges she faced. She reported feeling as though her “*eyes were darting around uncontrollably*,” making it impossible to form a cohesive picture of her environment. She noted that these darting eye movements were primarily horizontal and explained that she “*preferred horizontal lines*,” finding spaces without vertical lines “*much calmer.*” This real-time recording provided valuable insight into how Case 1 navigated cluttered spaces and highlighted the nature of her struggle to maintain visual coherence amidst the complexity, along with a resulting sense of stress and confusion.

**Figure 3:**
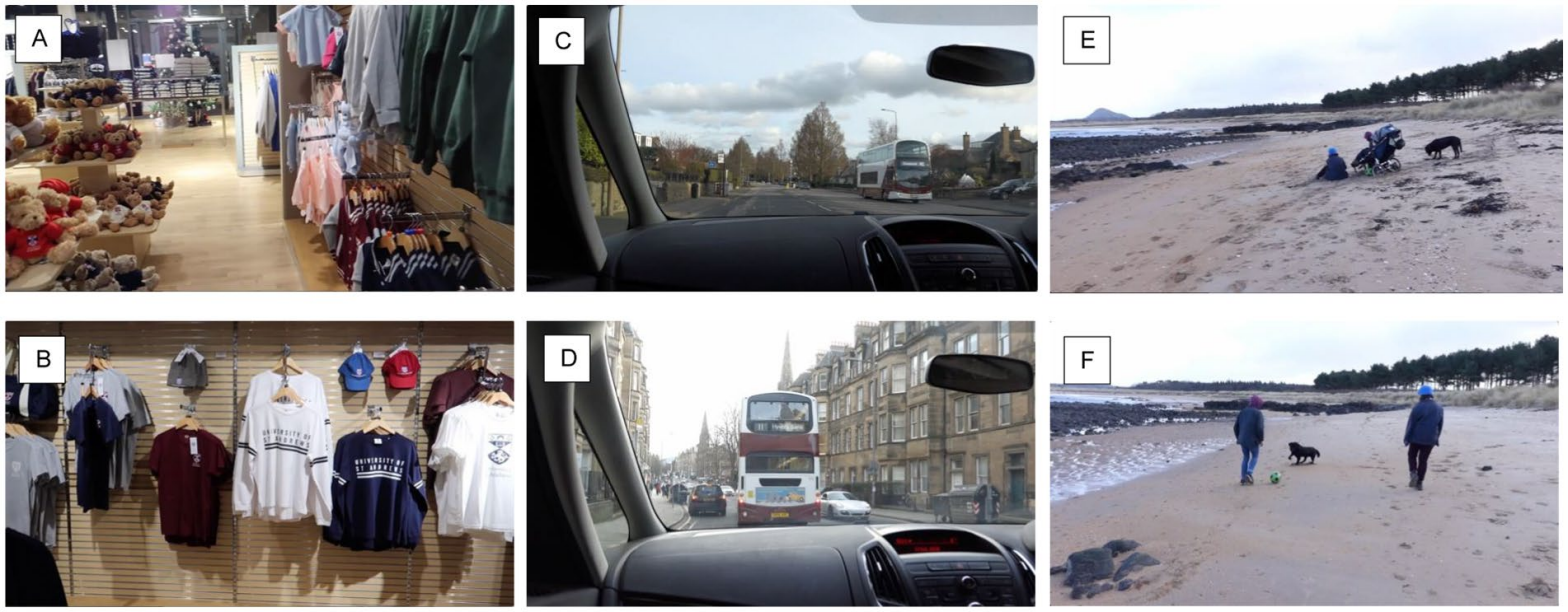
A representative still image from each video, showcasing the typical content and complexity level. The references **(A–F)** align with the video descriptions in Table 1.

To develop a video simulation of simultanagnosia, University of St Andrews Schools of Medicine and Computer Science partnered with the NGO CVI Scotland. Six short videos depicting everyday scenarios with varying degrees of complexity and movement, aiming to simulate simultanagnosia in a dynamic context, were created (Table 1 and Figure 3). The method of asking affected individuals to “say what you see” was once again employed to capture real-time descriptions of their visual experiences.

**Table 1:** The six videos used to create the simulations, along with their duration (in seconds) and a brief description of each.

Video title	Length (seconds)	Content
Shop (A)	69	Moving through a university gift shop, with toys, clothes and trinkets.
Shop (B)	32	Static position in the same shop, looking at shirts, moving towards the end, approached by someone.
Driving (C)	77	From the passenger seat position, moving (30 mph) through an open part of a town with parks and houses.
Driving (D)	44	From the passenger seat position, slow / stopped in traffic, build up.
Beach (E)	31	Walking on a beach, passing a small group having a picnic and some dogs playing.
Beach (F)	29	Walking on an open beach, passing some people playing football with a dog.

The references (A–F) correspond to the images labeled A to F in Figure 3.

**Figure 4:**
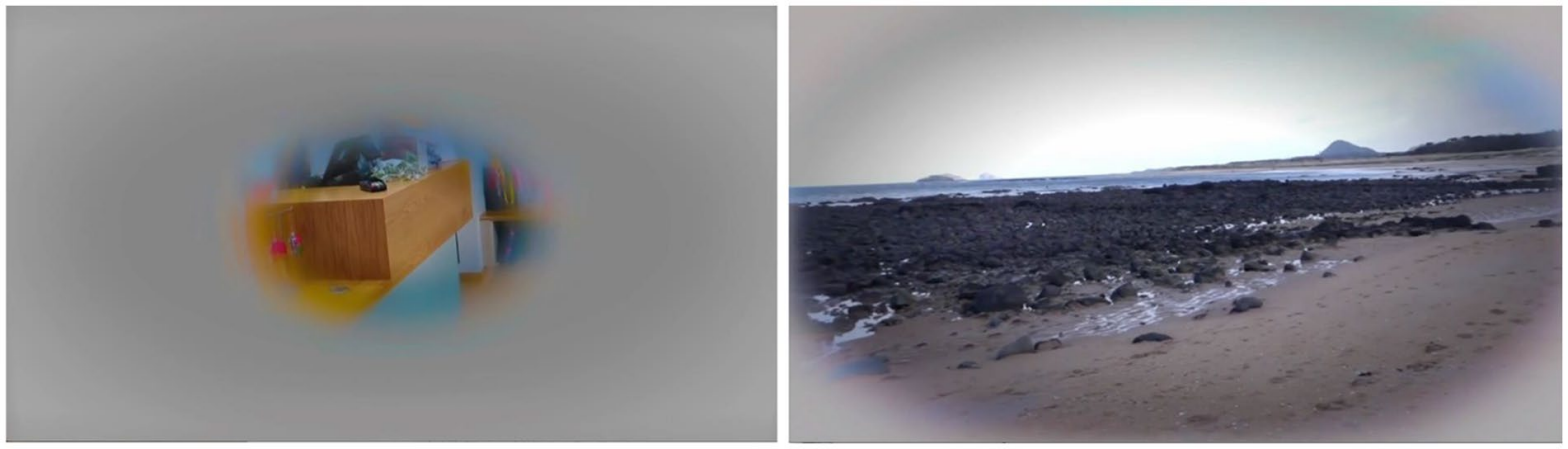
Images from the Shop (A) and Beach (C) videos with the added simultanagnosia simulation filter, showing the greater area of vision described on the open beach.

Case 1’s subjective visual challenges with visual crowding and search were initially explored via email. After viewing the videos, Case 1 provided written descriptions of what she was able to perceive. She found certain videos difficult to watch, at one point feeling startled. In one instance, she described seeing only part of a shop assistant’s smiling face, remarking, “*Cannot read expression”* while noting that the person, “*does not seem happy*.” During a beach video, Case 1 initially stated, “*I can see everything,*” but when a group of people having a picnic appeared, she fixated on that scene and could only see one dog, even though there were two. Once the film moved beyond the picnic, Case 1 remarked, “*Lovely open beach, I can even see the seagulls*,” which were small and in the far distance. The results aligned with Case 1’s previous experiences with photographs: as the visual complexity increased, she could perceive fewer items. To simulate this experience, a “fogging” filter was dynamically applied, to cover the areas of the image that had not been reported (Figure 4).

When the duplicate videos with the fog filter were sent to Case 1 for feedback, she commented, “*Some of the videos seem extreme, and I feel I can see much better than that. But then if I think about it and put myself in the different environments shown in the videos, they are pretty accurate*.” She particularly appreciated how the beach videos captured the constant fluctuation in her vision and felt the shop videos accurately depicted situations she had repeatedly experienced. Case 1 noted, “*I especially like how the filter highlights fast movement and how it reduces what you are seeing*.” A close family member, who had also viewed the videos, felt they accurately reflected the shifts in Case 1’s visual experiences, noting changes in anxiety levels and behavior in stressful environments, such as shops, compared to more open spaces like the beach. These findings suggested a dynamic element to simultanagnosia that, at the time, had neither been researched nor documented. The team were eager to share these novel insights with the broader CVI community but, mindful that the findings were based on one individual’s experience, sought input from two other adults with simultanagnosia as an element of their CVI diagnoses.

Cases 2 and 3 (Sections 4.2 and 4.3) were asked to watch the same videos in person, with their real-time responses being recorded for analysis. Both the volunteers were seated in quiet, uncluttered rooms and viewed the videos on a laptop positioned at eye level, 30 cm away. The videos were silent, and they were asked to naturally narrate what they could see without making any effort to search for details.

However, the findings from Cases 2 and 3 indicated that they may be more severely affected by simultanagnosia than Case 1, who appeared to perceive more, possibly due to years of practicing conscious vision strategies. Case 1 had not previously viewed the videos in the same “say what you see” manner as Cases 2 and 3, so she was asked to watch one of the videos in this way for comparison. With her agreement, this session was video recorded for review and analysis. The *Shop (A)* video was selected.

*Shop (A)*: At the start, the pace of the video was too fast for Case 1, and she physically pulled back from the screen, similarly to Case 3. Her vision became “stuck” on a teddy bear, and even after the video moved on, she was unaware that the teddy bears were no longer present. When later asked, “*When you cannot see things, what’s that experience like?*” she responded,

“*It’s almost like an anxiety response. I wanted to see what was there, yet I could not see it, so I got anxious about it. I got frustrated, and the emotions kicked in, which meant that I stopped looking at anything else. I could see the camera was still moving, but I was thinking about the teddy bears. I wasn’t responding to the information that was coming from my eyes… I wasn’t even aware of that. It’s almost like it had switched off that sense*.”

She found it challenging to stay focused on the task and had no memory of previously watching the video. Case 1 noted that she believed her visual function had improved over time, but was surprised by how difficult she found it to view the video. She explained that her fixation on the teddy bears was likely because she had liked teddy bears as a child. Interestingly, in this “live” review, she saw considerably less than she had during their original assessment of the video. The number of items correctly identified by Case 1 is documented alongside the results from Cases 2 and 3, with a comparative analysis presented in Table 2.

**Table 2:** Number of items identified by Cases 1–3 and the comparison across the six videos and one of the videos, Shop (A) with the simultanagnosia simulation filter added.

Number of items seen							
Video	Shop (A)	Shop (B)	Driving (C)	Driving (D)	Beach (E)	Beach (F)	Shop (A) + Filter
Length (sec)	69	32	77	44	31	29	69
CVI Case 1	6	–	–	–	–	–	–
CVI Case 2	5	6	8	6	9	8	5
CVI Case 3	7	5	13	5	8	6	5
Comparison	33	14	19	43	16	16	26

This illustrates the complexity of simultanagnosia and how personal emotional responses and past preferences can influence visual perception, especially in visually complex environments. It also suggests that even with conscious vision strategies, the dynamic nature of simultanagnosia continues to present significant challenges.

For the purposes of this manuscript, as Case 1 initially reviewed only one video, she agreed to view the remaining videos. In 2019, when Case 1 first viewed Shop (A) (Table 1), her performance did not stand out compared to Cases 2 and 3. However, upon re-watching the same videos 5 years later, the difference was significant (see Appendix 3). When Case 1 re-watched Shop (A) in 2024, she reported seeing 21 items, a substantial increase from the 6 items she noted in 2019. During this period, Case 1 continued practicing her conscious vision strategies, which may well have contributed to their improved visual perception. The fact that Case 1 had previously seen the video does not fully explain the 15 additional items she was able to identify, suggesting that these exercises have indeed been beneficial. While reviewing previous findings, Case 1’s experience of getting “stuck” on the teddy bears in Shop (A) was revisited, and the possibility of palinopsia was raised. Palinopsia is a phenomenon where visual images persist after the object has been removed from view (Bender and Feldman, 1976). Different types of palinopsia include seeing what was there before as either a vivid image, a “ghostly” translucent presence, or a trailing effect.

### 4.2 Case 2

Case 2 is an academically successful young man who is warm, friendly, and good-humored. He does not make eye contact during conversation because it is difficult for him, and he is unable to recognize faces. To navigate his surroundings, he has developed a self-taught visual search technique, scanning different points to mentally map his environment. Despite this, he often bumps into people or objects, not realizing they are there, and struggles to locate items that have been put away. Case 2 was asked to view the same videos as Case 1, and his responses are outlined below in the order he watched the videos. Table 2 provides the number of items he described in each video, and Appendix 3 contains his full verbatim responses.

*Beach (D)*: He identified two people, one dog (out of two), and a football, each when they appeared in the center of his gaze. He missed elements in the peripheral areas, such as a hill in the background and birds in the sky.

*Driving (E)*: He did not notice a large traffic light at the beginning but was aware of the traffic. Peripheral objects, including people, houses, trees, a wall, lamp posts, road signs, a bright blue bin, and additional traffic lights, were not observed. However, when trees and a large white building appeared in the center, they were recognized, though other nearby structures, including church spires, were missed.

*Shop (A)*: He noticed teddy bears and clothing but could not specify details, though he was able to read ‘University of St Andrews’ on a T-shirt. After 40 s, he requested the video be stopped, stating, “*It’s because it’s moving so much. I usually rely on looking at different points and making a mental map; it’s hard if it keeps moving all the time*.”

*Beach (C)*: He saw two people and recognized them as the same from the previous video. He missed a line of trees on the right and only noticed a hill when it briefly appeared in the center, saying, “*There appears to be a sort of mountain type object*.”

*Driving (F)*: He saw buses, cars, and one of three church spires in the center, along with traffic lights, but nothing in the periphery. People, shops, buildings, and trees on either side were missed.

*Shop (B)*: Similar to the Shop (1), he recognized clothing but could not specify details, though he again read ‘University of St Andrews’ on a T-shirt. Despite a pause, he missed clothes hangers in the center of the screen. He noticed the assistant’s torso briefly on the side but missed a woman in the center, a man behind her, and the assistant gesturing for attention.

Next, he was asked to view *Shop (A)* again with the simultanagnosia simulation filter. He approached it as before, narrating what he saw.

*Shop (A) with Simultanagnosia Simulation Filter*: He identified bears, clothes, and later additional items, such as the cash register. With the filter, he was able to watch the entire video, remarking, “*I can see everything.*” When asked to compare the filtered and unfiltered versions (Figure 4), he said, “*not that much difference to be honest*.” Upon pausing the video at the end, he acknowledged the grey fogging effect, noting, “*my mind maps images, so at some point during filming I’ve looked away from the center … there is a large grey color that cut out a large portion of the screen*.” He confirmed he only noticed the filter when looking away from the center, and when asked to focus on the paused image, he said he could see “*a bit of it [the grey fog] on the edges when I look at the center, but only slightly*.”

**Figure 5:**
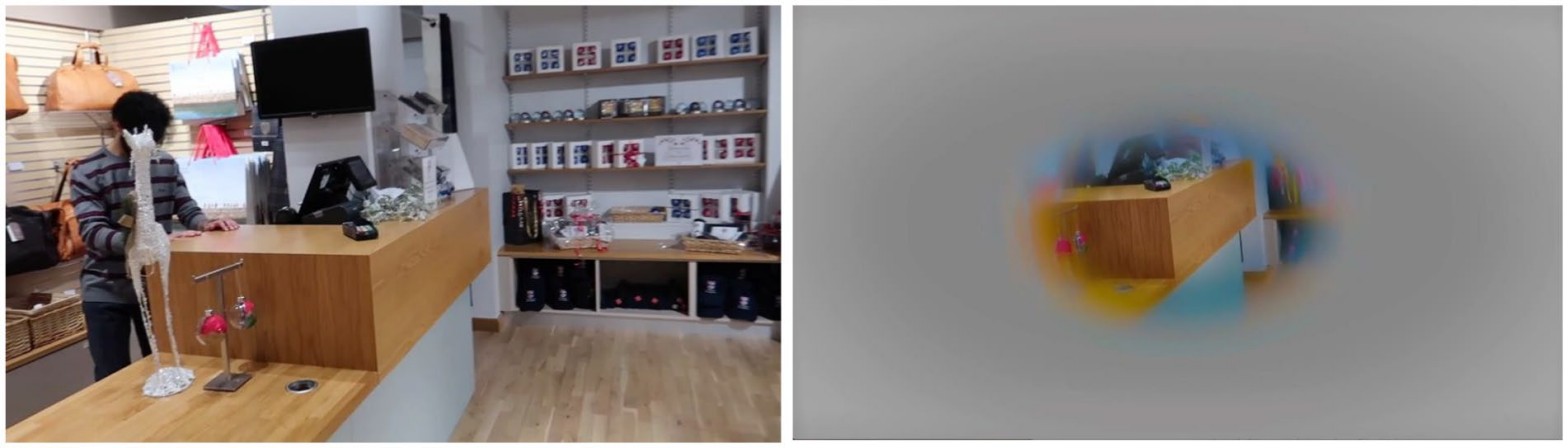
The right image is the still image from the end of the Shop (A) video. The right image is the same picture with the filter applied.

These observations reinforce the dynamic nature of his visual experience and how simultanagnosia affects his ability to perceive complex scenes (see Figure 5).

When asked how he was feeling, Case 2 responded, “*I am feeling slightly mentally fatigued*.” Upon explaining that their descriptions suggested fluctuations in the area of his field of vision, he reflected, “*I’m not sure if I just do not notice it, but I do not tend to notice my field of view changing. I think that’s because it’s sort of gradual if it does… I do not notice it* var*ying*.” This response suggests that while there may be dynamic changes in his field of vision, these shifts are not readily perceptible to him, even though the findings indicate variations based on visual complexity.

### 4.3 Case 3

Case 3, a warm, communicative, and intelligent young woman, struggles socially in crowded environments and quickly becomes tired and overwhelmed by excessive visual complexity and noise. Busy places cause significant discomfort, and crossing roads safely presents challenges. She relies heavily on familiar routines and environments she has memorized for navigation. Case 3 was asked to view the same videos as Cases 1 and 2. Her responses to each video are outlined below, with additional discussion points. The number of items described in each video is listed in Table 2, and their full verbatim responses are included in Appendix 3.

*Beach (D)*: Case 3 identified two people, two dogs, and a green football. However, she did not notice elements in the peripheral areas, such as the hill in the center left or the trees in the center right.

**Figure 6:**
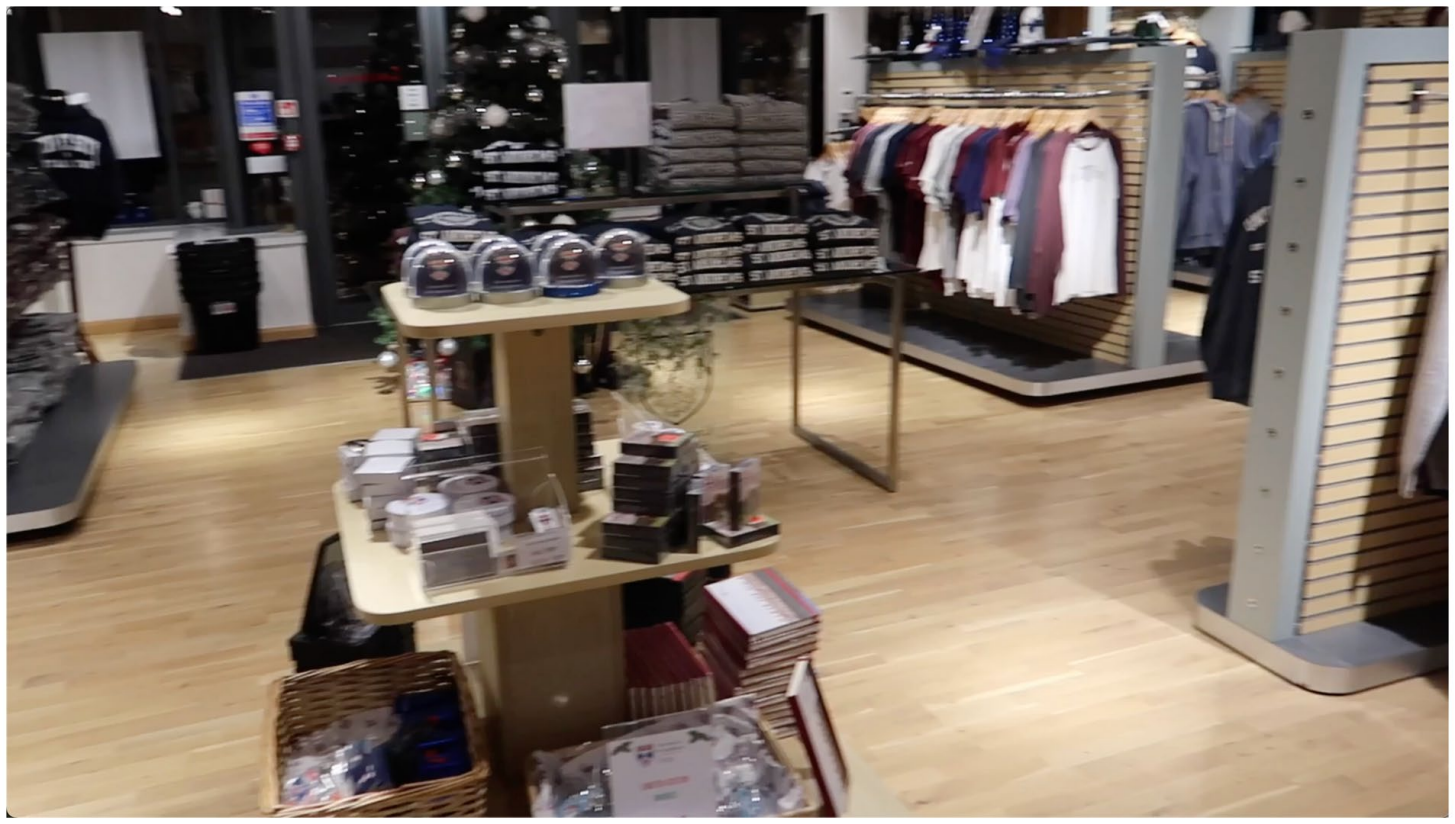
This still image, displayed on a laptop screen, was sufficient to provoke a startle response in Case 3, who grimaced and instinctively moved their head away from the screen. Despite the slight ‘softening’ effect of the blur, the image remained difficult for Case 3 to visually process, as indicated by her reaction.

*Shop (A)*: As the video was being set up, a slightly blurred, still image of shop displays appeared on the screen (Figure 6). Upon seeing this image, Case 3 startled, grimaced, said “*ugh*” and moved her head away from the screen. This reaction mirrored a similar response from Case 1, who, when viewing a photo of supermarket displays, commented, “*My first response was to pull away from the photo, as it was too much information, and my automatic reaction was to try to get away from it*” (University of St Andrews, 2024).

Once the video started, Case 3 described seeing teddy bears and children’s clothing, though she could not specify details. After long pauses, she later mentioned seeing more clothes (unspecified), the tills, and the sales assistant. These reactions suggest that visual complexity, particularly in cluttered environments like shops, can be overwhelming and cause a strong aversive response, reinforcing the need for carefully managed environments to reduce visual overload in individuals with simultanagnosia.

*Driving (E)*: Case 3 noticed the traffic lights at the beginning of the video but missed houses, trees, and cars on the sides, though she did observe the bus as it moved into the center of her field of view. She also reported seeing moving cars and a person with a dog to the side but failed to notice another person in the same position a few seconds later. After a pause, she noticed a pink van and, towards the end, some houses and the traffic lights again. Aside from the person and dog, Case 3 did not perceive anything in the peripheral areas.

*Beach (A)*: Case 3 reported seeing rocks, sand, a black Labrador dog, a wheelchair, one person (though there were two), a red setter dog, some trees, and the sea. However, she did not notice the hill in the background. These observations further highlight Case 3’s challenges in perceiving peripheral details and their tendency to focus on central elements, missing key objects or people in more complex scenes.

*Driving (F)*: Case 3 observed one double-decker bus, though there were two next to each other, and noticed a bright blue car on the far left, which stood out due to its color. She mentioned “some buildings” and “traffic” but was not specific. She missed key elements such as people, three church spires, a bus stop, shops, and traffic lights on both the left and right of the screen. Across all videos, the only peripheral items Case 3 noticed were a person and a dog in *Driving (E)* and the blue van in *Driving (F)*. When asked, Case 3 mentioned she was a dog lover and is particularly drawn to bright colors, especially pink. After viewing this video, she requested a break that lasted 15-min before continuing.

*Shop (B)*: Case 3 noticed t-shirts and bright red and blue baseball caps but missed grey and navy-blue caps on either side. She also did not see a shirt being removed, the assistant walking towards the camera and gesticulating, or three people in the shop, including the assistant, during a second appearance. These responses emphasize how personal preferences, like a fondness for dogs and bright colors, can influence what Case 3 perceives in cluttered, complex environments. It also highlights her difficulty in noticing more neutral or less distinct items in the visual periphery.

Following this, as with Case 2, Case 3 was invited to view the simultanagnosia simulations the team had been developing, and she agreed (Figure 4).

*Beach (D) with Simultanagnosia Simulation Filter*: Case 3 saw more background elements (rocks, sand, and trees) that had not been visible previously. She also recognized the dog, two people, another dog, and a green ball as before, but still did not notice the hill. When asked about the filter, Case 3 commented that the video felt “more narrowed down” but that she was “not really aware there was less to see.” In the discussion afterward, Case 3 remarked that with the filter, it seemed like there was more to see, not less.

*Shop (A) with Simultanagnosia Simulation Filter*: Although Case 3 did not notice many additional items (mentioning a teddy bear, some baby clothes, a pink jumper, some displays, and a till), she commented that the scene appeared less cluttered with the filter.

*Shop (B) with Simultanagnosia Simulation Filter*: Case 3 was able to read ‘University of St Andrews’ on a top when viewing the video with the filter. Without the filter, she could only see “t-shirts” in the same video.

### 4.4 Comparison (adult without CVI)

Although it was clear that Cases 1–3 were experiencing visual perception far from typically, the definition of ‘typical’ vision had not yet been established for comparison. To address this, one of the team members, who has no visual impairments and had not previously seen the videos, agreed to watch the same videos in the “say what you see” manner for comparison. They also gave permission for the session to be video recorded for review and documentation purposes. This provided the team with an initial reference point for typical visual perception under similar conditions, helping to further explore and contrast the unique visual challenges experienced by individuals with simultanagnosia.

Table 2 presents the number of distinct items correctly identified by Case 1, Case 2, Case 3, and the comparison, across six videos, as well as one video with the simultanagnosia simulation filter. This comparison provides a quantitative measure of the differences in visual perception between the cases and someone with typical vision. The full verbatim responses from the comparison participant are available in Appendix 3 for detailed reference. This table helps highlight how each individual’s visual experience differs, particularly in complex or cluttered environments, and provides a clearer understanding of how simultanagnosia affects visual recognition and naming of objects.

At this stage, it was deemed inappropriate to ask anyone else to view the videos without obtaining ethical clearance, given that the content could be stressful and challenging for viewers. However, a small internal team, including another adult with simultanagnosia and a parent, was asked for feedback, and they confirmed that the findings resonated with their own experiences.

## 5 Discussion

The initial aim of the project was to explore the feasibility of accurately simulating the visual perception challenges associated with DSD, particularly in relation to simultanagnosia. These difficulties have been characterized by problems such as locating familiar objects, like identifying a known individual in a crowd or finding a toy in a toybox (Bennett et al., 2018, 2020). It became evident that, while participants could only name one object at a time, when viewing videos with less visual clutter, they could perceive a broader area of the scene. By contrast, in more complex environments, such as the *Shop (A)* video, the area of vision where they could see appeared to be significantly reduced. This limitation made it more challenging to locate and identify familiar objects, resulting in prolonged task completion times, along with increased fatigue and stress.

Visual neglect, a condition marked by a lack of visual attention in areas where there is typically a normal visual field, has been described as ‘a neurological disorder characterized by a deficit in attention to stimuli on one side of the body’ (Ting et al., 2011). This condition is most commonly associated with one-sided impairment following a stroke (Linden et al., 2005). Zihl and Dutton (2015) further elaborate that “after acquired brain injury, the field of attention can be altered in an even more dramatic way, with part of the field of attention potentially missing.”

In developing the simulations, the observed visual areas suggest a form of attentional visual field loss, though affecting the periphery rather than one side. Additionally, the extent of peripheral attentional field loss appeared to fluctuate for the same individual, depending on the visual complexity of the video, indicating that this type of attentional visual field loss is dynamic. The negative impact of visual complexity on individuals with CVI is now well-documented. For instance, Bennett et al. (2018) described the difficulty in finding a toy in a toybox when distractions were added, and McDowell and Butler (2023) found that increasing complexity made shape-matching tasks more challenging. Both studies demonstrated that children with CVI struggled significantly more with these tasks than control groups. Moreover, the three case studies consistently showed that reducing visual complexity not only improved access to the visual field but also fostered greater emotional comfort. This aligns with recent findings (McDowell, 2023) and suggests that visual difficulties associated with DSD can be either exacerbated or alleviated by changes in environmental complexity (McDowell and Butler, 2023).

The concept of a simultanagnostic ‘window’ of vision within the attentional dynamic peripheral visual field can be likened to a camera’s shutter-controlled aperture, dynamically opening across the field of vision. A similar phenomenon has been previously described. Holmes (1918), in his account of Case 2, observed that the patient ‘could not be certain of the position in space of objects which he could see’ and experienced ‘bilateral inferior hemianopia,’ suggesting optic ataxia and potential visual neglect in the lower visual field. This implies that Case 2 may have had Balint Syndrome, a severe form of DSD (Gillen and Dutton, 2003). Holmes also noted that ‘the borders of his blind portion were indefinite,’ which could be explained by the variability of this ‘window’. Milner and Goodale (2010) described ‘central sparing’ where a small window of vision is available in the center of the visual field, in patients with optic ataxia. They suggested that this might occur because, in the absence of a functioning dorsal stream, the ventral stream compensates, relying on central vision. They observed “some cases of central ‘sparing’ during real-time reaching in optic ataxia patients.” It raises the question of whether this central sparing might instead represent the simultanagnostic window of vision reported by Cases 1–3.

Zihl and Dutton (2015) similarly observed in Balint Syndrome that ‘bilateral posterior brain injury can cause bilateral restriction of the field of attention, sparing, depending on the degree of severity, only a small area for visual information processing.’ This phenomenon may also align with the concept of the ‘window’ of vision. Jacobson et al. (2002) found that in children born prematurely with bilateral posterior parietal brain injury, some had normal outer limits of the peripheral visual field but struggled with peripheral attention in crowded environments, consistent with our case study findings. Most recently, Hokken et al. (2024) used eye-tracking to map the gaze of CVI participants in increasingly complex scenarios, with findings consistent with our observations. In our study, while the ‘window’ of vision was predominantly central, it occasionally extended, particularly when a stimulus ‘popped out.’ For example, Case 3, a dog lover, was automatically drawn to a dog in the periphery. This ‘pop-out’ effect, where attention is involuntarily drawn to a particular visual stimulus amid competing stimuli, has been previously described (Adler and Orprecio, 2006).

When asked about the ‘shape’ of their window of vision, Case 1 explained that it matched the shape of whatever she was directly observing. For instance, when looking at a teddy bear, the window was teddy bear-shaped, rather than a circle with the bear in the center. She could only see the teddy bear itself, with nothing visible around it. Similarly, when looking at her splayed hand, she only saw the hand, with no perception of the space around it or between the fingers.

**Figure 7:**
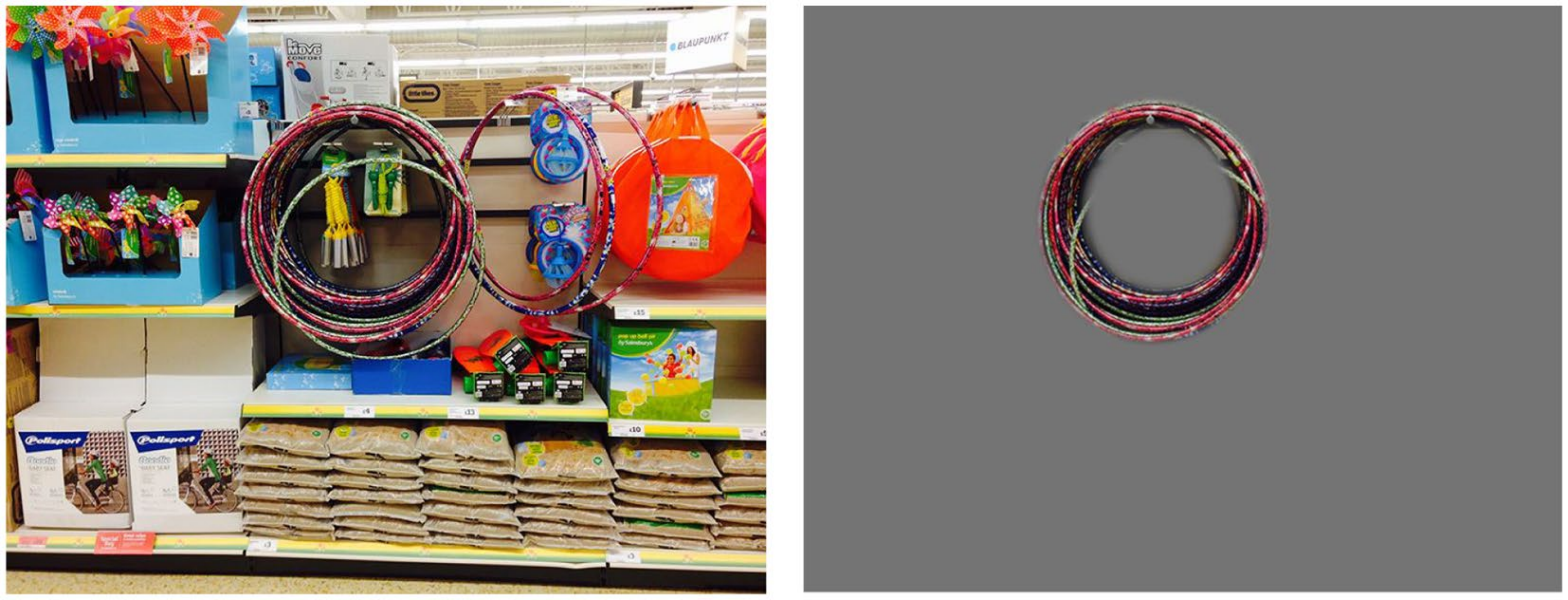
Photo of hoops with what was not seen removed, including the objects in the center of the hoops, suggesting their window of vision in this instance was ‘hoop shaped’.

Figure 7 illustrates how Case 1 perceived the hoops: she not only failed to notice the surrounding environment but also overlooked the toys hanging within the center of the hoops. This observation suggests that our initial simulations, which used circular and oval “windows” to represent the visual experience, may require refinement to more accurately capture the true nature of their vision. Notably, this differs from Figure 2, reflecting the evolution of our understanding for the ongoing refinement of our simulations.

Case 1 described her experience when viewing the display with the hoops, noting, “*If I tried to move my gaze away, it quickly got drawn back to them. A bit like a magnet*.” This observation resonates with findings by Jackson et al. (2009), who identified a ‘competing’ element in a patient with simultanagnosia. Their patient struggled significantly more when multiple letters were presented simultaneously, compared to when letters were shown sequentially and overlapped, even for brief periods. It raises the question of whether Case 1’s difficulty in shifting gaze from the hoops could represent a more extreme form of competing simultanagnosia, where the competing elements are so overpowering that they become visually disabling. This could also offer insight into why some profoundly disabled children with CVI are drawn to stare at bright lights (CVI Scotland, 2024a). If this is indeed a manifestation of competing simultanagnosia, it may serve as a potential method to identify the condition in individuals who may not have the capacity to follow instructions or answer questions, offering a new diagnostic tool for this population.

Cases 1–3 all found the shop videos, particularly *Shop (A)*, to be the most challenging, causing significant stress and fatigue. Case 2 had to stop *Shop (A)* at 40 s for a break, suggesting that environmental complexity affects not only visual processing but also overall well-being. This observation aligns with McDowell’s (2023) study, where children with suspected CVI reported anxiety and difficulty regulating emotions in complex environments. It remains unclear whether emotional states, such as stress or anticipating a difficult situation, further impair visual performance. The need for breaks in Cases 2 and 3 suggests that both the complexity of the environment and emotional factors may influence visual functioning.

The responses to the driving video were notable. Despite the complexity of the environment and the speed of movement, which paralleled the challenges of the shop videos, none of the volunteers reported the same level of fatigue. The muted colors in the driving video may have contributed, but it is also possible that the simulation of being in the car with someone else driving allowed the participants to relax, as their automatic alert systems may have been temporarily suspended. This aligns with the idea that emotional state plays a key role in optimizing visual experiences. Further research is needed to determine whether reduced stress improves visual performance, but the findings suggest that challenging environments may be easier to tolerate when someone else is in control, such as being pushed in a wheelchair through a busy airport. This could also explain the popularity of activities like horse riding for individuals with CVI (CVI Scotland, 2024b).

When asked about her experiences during silent periods, Case 3 responded with “*I do not know*,” while Case 1 described it as if her ‘vision sense’ had switched off. This highlights the paradox of anosognosia, where individuals are often unaware of their own impairments and view them as normal, rendering the condition disabling yet asymptomatic. However, in this study, Cases 1–3 were able to articulate their difficulties because they had received detailed explanations of their visual impairments years earlier, allowing them to recognize and navigate their challenges.

When viewing the video with a ‘fog’ filter, all three with DSD found the experience much easier. Case 3 described the fogged video as less cluttered, although she was unaware of the fog itself, which covered over 80% of the screen at times. The number of items she could see remained nearly the same (Case 2: 5 items with and without the filter; Case 3: 7 items without the filter, 5 with), but the task was perceived as less demanding, suggesting that reducing visual complexity alleviates demand on the brain.

Hypothesizing the mechanism behind these observations, we can conceptualize each visual nerve fiber as contributing a voxel; numerous smaller voxels in the central visual field and fewer, larger ones in the periphery. As the demands on the posterior parietal mapping system exceed its limited capacity, the smaller central fibers may continue functioning, while the larger peripheral fibers may temporarily shut down, possibly as a mental compensatory strategy. This results in progressively less peripheral visual information reaching the brain as clutter increases, potentially rendering the individual unable to perceive anything meaningful. This could explain the dynamic narrowing of the attentional visual field, the ‘pop-out’ effect beyond the central visual area, and why adding a filter reduces visual stress by lowering the system’s cognitive load. Given that reducing visual input through a fog-like filter appears to lessen the demand on the mapping system, it is worth considering whether spectacles with peripheral masking could potentially help children with CVI. This approach could be particularly useful in low-movement environments, such as classrooms or while watching television, where unnecessary visual clutter could be minimized, thereby reducing the negative impact of incoming unnecessary clutter and complexity.

The challenges observed in these case studies, along with findings from Hokken et al. (2024), suggest that the peripheral field of vision in individuals with simultanagnosia is dynamic, often significantly impairing vision, as demonstrated by all three cases. This dynamic nature possibly affects the formation of visual memories. For instance, Case 2’s inability to recognize faces or facial expressions is a common issue in CVI (Bauer et al., 2023). This difficulty may stem from an inability to perceive an entire face, as many children with CVI anecdotally report that they can only see parts of a face. Further research is required to better understand this phenomenon. This differs from prosopagnosia, a facial recognition disorder resulting from temporal lobe dysfunction, which is typically associated with ventral stream impairment. Although prosopagnosia and CVI-related facial recognition difficulties may co-occur (Thomas et al., 2009). Simultanagnosia and its potential complexities might contribute to facial recognition deficits through mechanisms distinct from those involved in ventral stream dysfunction. Prosopagnosia has also been reported in cases of Balint syndrome (Rosselli et al., 2001), suggesting overlapping but not necessarily identical patterns of impairment.

Deficits in encoding the spatial coordinates of facial features in developmental prosopagnosia (Barton et al., 2003) bear similarities to the deficits reported for simultanagnosia concerning the binding of visual attributes of objects (Robertson et al., 1997). However, it is important to recognize that these similarities do not necessarily imply direct causality between dorsal stream dysfunction and ventral stream processes. Rather, these deficits may reflect challenges in inter-stream connectivity and integration within broader visual processing networks, which involve both vertical and horizontal connections (Robertson et al., 1997).

While simultanagnosia may contribute to impairments in facial recognition through disruptions in visual attention and spatial integration, current evidence does not support a direct influence of dorsal stream dysfunction on ventral stream function. This underscores the need for further research to elucidate how distinct visual pathways interact and compensate for each other, particularly in cases where processing within a specific region deviates from typical functioning. Future studies focusing on the interaction between spatial and object pathways could offer insights into the neural mechanisms underlying these co-occurring deficits.

In clinical practise, CVI can be associated with isolated pathology within the posterior parietal lobes, while the temporal lobes remain unaffected. This pattern suggests the possibility of isolated DSD. For instance, in their review, Dutton and Jacobson (2001) describe how lesions in the posterior parietal lobes can lead to deficits in spatial processing and consequently, visual attention, without impairing ventral stream functions such as object recognition. Similarly, Pehere et al. (2018) in their overview of the neural pathways involved in CVI, also describe instances in which parietal lobe damage was associated with deficits in spatial awareness and visual attention, while also sparing ventral stream functions. Michel and Henaff (2004) further explore simultanagnosia resulting from posterior parietal lesions, demonstrating that such damage disrupts the ability to perceive multiple objects simultaneously, a key function of the dorsal stream, while preserving ventral stream capabilities like object recognition. In contradistinction, persistent ability to see movement and move accurately through three dimensional space, despite bilateral loss of the occipital and temporal lobes (the Riddoch phenomenon), potentially highlights the nature of dorsal stream function when the middle temporal lobes (serving movement perception) and posterior parietal lobes remains intact (Arcaro et al., 2019). These studies underscore a clear distinction between the roles of the dorsal and ventral streams in visual processing. Damage specific to the posterior parietal lobes can lead to impairments in spatial and attentional processing while preserving ventral stream functions and vice versa. Understanding this distinction is crucial for accurately identifying and supporting individuals with CVI, particularly those with isolated DSD, as it provides a foundation for more tailored interventions based on the type and location of the underlying neural damage or dysfunction.

Understanding simultanagnosia reveals it to be a highly nuanced and individualized condition. One emerging insight is the role of the ‘pop-out’ effect, which may be influenced by personal preferences. For example, Case 1 was particularly drawn to teddy bears and the color blue, while Case 3 preferred bright colors like pink and had a strong affinity for dogs. This suggests that recognizing the individual’s preferences may offer opportunities to minimize distracting “pop-out” stimuli or, conversely, leverage them to aid in locating objects. Research from Hokken et al. (2024), using eye-tracking technology, further connects the adverse effects of environmental complexity to the dynamic nature of the attentional visual field in simultanagnosia. By testing vision with specific targets rather than relying on naturalistic descriptions, as seen in tests like the Birthday Party Test (de Vries et al., 2022), the Hokken et al. approach offers a more controlled way to study these visual processing challenges. Combining both approaches, targeted and naturalistic, may provide deeper insights into the complexities of simultanagnosia.

In 2020, the University of St Andrews and CVI Scotland shared some of the findings, including the video *Beach (C),* along with a carefully explained description of the exploratory nature of the findings and their limitations. A short video summarizing the findings was also part of this resource (University of St Andrews, 2024). The public’s response to this material, was overwhelmingly positive. The short video, in particular, seemed to resonate with many viewers, including adults who identified CVI in themselves after watching it. Examples of community feedback included: “*OMG! You nailed it! That’s exactly what it’s like. Bravo!*,” “*At last, I can show everyone my child’s world so they can understand better… now I can show them!!!*,” “*LOOK!!! …this is what it would feel like to walk in my shoes*.” The success of this process laid the groundwork for a subsequent clinical trial to test these theories, including the creation of a control group.

Currently, there are no standardized measurements for simultanagnosia or other higher visual processing difficulties associated with CVI, despite the likelihood that the severity of these impairments varies between individuals. It may be feasible to develop measurements targeting the various elements that comprise simultanagnosia. For example, the “window of vision,” which appears to be dynamic, could be quantified as a percentage of the total visual field, potentially capturing its range from largest to smallest. Such measurements could allow for tracking improvements over time. In the case of Case 1, vision seemed to improve through their ‘conscious vision strategies’, but it remains unclear whether the visual field itself expanded, she better tolerated difficulty, or gained more control over the dynamic aspects or ‘pop-out’ effects.

An inability to locate a pointed-out target can manifest as a child appearing unable to move their eyes toward it, a condition known as apraxia of gaze, an element of dorsal stream dysfunction (DSD). Traditionally, apraxia of gaze and simultanagnosia have been described as separate components of DSD. However, considering findings from studies where individuals were asked to identify a target in a visual scene (Bennett et al., 2018, 2020; Hokken et al., 2024), it is plausible that apraxia of gaze may be a consequence of simultanagnosia rather than a distinct visual impairment. If a person is unable to perceive a target, they cannot move their gaze toward it. This could explain instances where gaze becomes ‘stuck,’ as seen with Case 1 and the hoops, suggesting that apraxia of gaze might be an effect of simultanagnosia’s interference with visual processing, rather than a separate condition.

Identifying the underlying cause of these difficulties is crucial to ensuring that interventions target the cause of the visual impairments rather than its symptoms. Identifying the underlying cause of these difficulties is crucial to ensuring that interventions target the cause of the visual impairments rather than just their symptoms. A CVI question inventory, validated by MacIntyre-Beon et al. (2012) and recently described using the acronym “CVI-I” for Cerebral Visual Impairment Inventory by Chandna et al. (2024), was designed to capture a range of visual behaviors associated with CVI in children.

Although the 2012 paper does not explicitly differentiate between types of CVI, the inventory provides insights into various CVI-related visual processing challenges, which have helped clinicians recognize different profiles of CVI presentations in practice.

Without concrete metrics, it is difficult to assess progress or identify where adjustments to support strategies are needed. Although the Hellgren et al. (2020) study identified visual perceptual challenges in preterm children rather than specifically diagnosing CVI, it used a control group and the CVI-I (MacIntyre-Beon et al., 2012; Chandna et al., 2024) to compare visual function across 39 areas. This approach could be adapted to develop percentage measurements for simultanagnosia, allowing for better differentiation of symptoms and treatment outcomes. Recent work by Hay et al. (2020) extended this methodology, demonstrating a direct correlation between total inventory scores and standard VMI scores for a subset of children with CVI followed over 6 years. This study used age-inappropriate configural disruption in copied drawings as evidence of “local capture” (Dalrymple et al., 2007, 2009; Karnath et al., 2000) and employed VMI standard scores to quantify the severity of simultanagnosia. Furthermore, studies specifically focusing on CVI diagnoses using validated inventories provide valuable insights. For instance, Ortibus et al. (2011) examined the screening value of a CVI questionnaire, while van Genderen et al. (2012) focused on diagnosing CVI in children with good visual acuity. Tsirka et al. (2020) explored the clinical application of the Insight Inventory in CVI and its effectiveness in guiding tailored habilitation strategies. New findings from Chandna et al. (2021) emphasized higher visual function deficits in children with CVI who have good visual acuity, a theme further explored in their 2024 study, which highlighted that these deficits can manifest independently of visual acuity measures (Chandna et al., 2024). These studies collectively underscore the need for validated tools to accurately identify and address the diverse presentations of CVI, particularly in cases where symptoms are not solely linked to reduced visual acuity.

## 6 Conclusion

The case studies presented in this work highlight the dynamic and individualized nature of dorsal stream dysfunction (DSD) in individuals with cerebral visual impairment (CVI). Our findings suggest that the symptoms of simultanagnosia extend beyond the traditionally understood limitation of perceiving one or two objects at a time. Instead, we observed a dynamic constriction of the attentional visual field, which fluctuates based on the complexity of the environment. This nuanced understanding of simultanagnostic vision underscores the need for a more refined approach to both diagnosis and intervention.

The concept of a dynamic “window” of visual attention, influenced by both internal factors (such as anxiety) and external complexity, presents new avenues for targeted therapeutic strategies. Interventions that reduce visual clutter and complexity, or leverage personal preferences, may help optimize visual functioning and reduce fatigue. These insights could be particularly beneficial for children at sensitive developmental stages, where early and effective interventions are crucial.

Furthermore, our findings highlight the importance of exploring how different visual processing streams in the brain interconnect and compensate for each other. Understanding these interactions may provide deeper insights into the variability of CVI symptoms and open pathways for developing more tailored support strategies. Future research should focus on the neural mechanisms underlying these dynamic visual experiences and explore potential interventions, such as adaptive visual filters or environmental modifications, to enhance quality of life for individuals with DSD.

## Data Availability

The original contributions presented in the study are included in the article/Supplementary material, further inquiries can be directed to the corresponding author.
