# Supplementary material for: Emulation of the subjective experience of visual dorsal stream dysfunction: a description of three in depth case studies
Source: Front Hum Neurosci. 2025 Jan 6;18:1496811. doi: 10.3389/fnhum.2024.1496811 (PMC11743676; doi:10.3389/fnhum.2024.1496811)
Supplement: Supplementary file 1 [file Data_Sheet_1.docx]

**Appendix 1: Explanation of Conscious Vision Strategies**

**First-hand account kindly provided by Case 1.**

The concept of "conscious vision" emerged from my journey to address the challenges associated with unconscious visual functions due to CVI (Cerebral Visual Impairment) and DSD (Dorsal Stream Dysfunction). Initially, when ophthalmologist Professor Gordon Dutton explained that my visual difficulties were unconscious functions, it made sense why neither I nor anyone else had identified them for nearly two decades. Professor Dutton suggested that I needed to strive to render these unconscious functions conscious. However, at that time, there were no specific strategies to achieve this, particularly for DSD, so I had to devise my own approach.

Around this time, I was also exploring mindfulness to alleviate anxiety related to my visual difficulties. Mindfulness emphasizes being present in the moment, often through conscious awareness of one’s surroundings or breath. I learned a technique called "conscious breathing," which involves counting breaths to focus attention. This inspired me to apply a similar technique to my vision.

**Developing Conscious Vision Techniques**

I began practicing conscious vision in simple visual environments, gradually progressing to more challenging ones as my skills improved. The essence of conscious vision involves deliberately focusing on what my visual system is doing during various activities. For example, when speaking with someone, I made a conscious effort to maintain eye contact, knowing my gaze would otherwise drift. When walking, I focused on keeping my vision steady and aware of my surroundings.

Over time, I developed specific strategies, such as the "wagon wheel strategy," which we have previously discussed. The core principle is that I am actively forcing my visual system to perform functions that are typically automatic for others. This requires constant concentration, focus, and dedication.

**Empowerment Through Knowledge**

The only reason I was able to develop these strategies was because I was empowered with knowledge. Without the explanation of my unconscious visual functions, I would not have known where to start. This experience has been transformative, allowing me to gain greater control over my vision.

**Parallels with Mindfulness Techniques**

Interestingly, this approach aligns with basic mindfulness practices. Techniques like the "body scan" or the exercise of noticing five things you can hear, see, smell, taste, and feel, encourage conscious awareness of sensory inputs. Similarly, conscious vision involves deliberately focusing my awareness on my visual processing, to better understand and control what my visual system is doing.
