# Supplementary material for: Emulation of the subjective experience of visual dorsal stream dysfunction: a description of three in depth case studies
Source: Front Hum Neurosci. 2025 Jan 6;18:1496811. doi: 10.3389/fnhum.2024.1496811 (PMC11743676; doi:10.3389/fnhum.2024.1496811)
Supplement: Supplementary file 2 [file Data_Sheet_2.pdf]

Visual and motor deterioration in Asperger's syndrome- a case report

I Hay[1],M.Nassar[2], S Biggar[3],
[1]NHS Dumfries and Galloway, UK; [2]NHS Dumfries and Galloway, UK & Minia University, Egypt; ; [3]Dumfries and Galloway Council, UK.

NHS Dumfries & Galloway

Introduction

Autism spectrum disorders (ASD) are a heterogeneous group of behaviourally defined neurodevelopmental disorders which have in common core deficits of severe impairment in reciprocal social interaction, communication and imagination, together with repetitive/inflexible behaviours[1,2]. Reciprocal social interaction requires competent vision perception and may be impaired in children with cerebral visual impairment[3] (CVI) as well as ASD. Motor skills depend heavily on vision, are commonly impaired in autism[4] and "dysfunction may be a fundamental component of the ASDs"[4].
The neurological basis for the motor impairments present in ASD remains unknown.

The posterior parietal area links to the visual cortex by the dorsal stream pathway (Fig.1). It creates an unconscious three-dimensional virtual map of the surroundings, affording the coordinates for visual guidance of movement amongst surrounding items. Bilateral dysfunction of the parieto-occipital cortex, linked to the visual cortex by the dorsal stream pathway, variably produces a triad of impairments: simultanagnosia, optic ataxia, and gaze apraxia. This disorder, rarely reported in childhood, comprises 'Balint's syndrome'(Fig.2). Adults with Balint's syndrome (BS) have severe visuomotor impairment with behaviour suggesting blindness, despite normal eyesight.

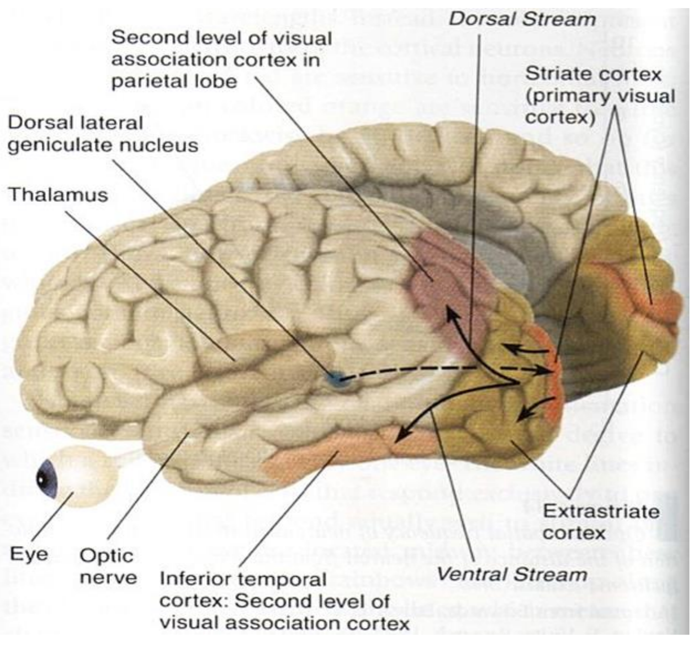

Fig.1: Higher order visual processing pathways

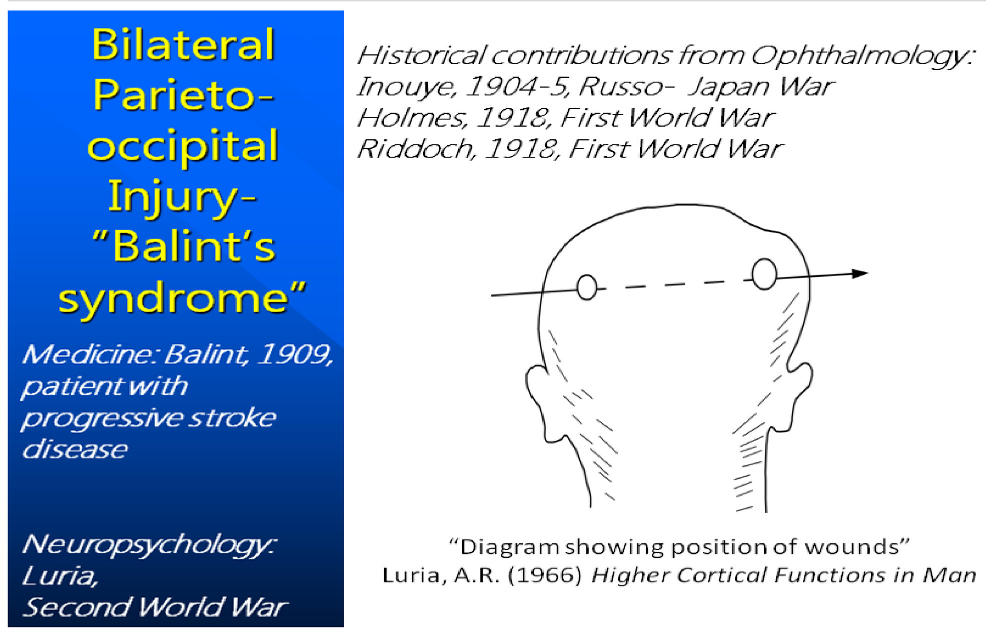

Fig 2: Sources of earliest descriptions of Balint's syndrome

Simultanagnosia reduces the visual attentional window, producing piecemeal perception. Optic ataxia (OA) produces a wandering, wide grasp dependent on tactile-proprioceptive feedback. Case reports of BS in the paediatric population are few[5,6]. The frequency of the condition is unknown; the diagnosis may be missed without specific examination for the Balint triad. The term 'dorsal stream dysfunction' (DSD) describes milder degrees of the disorder. **We report a case of progressive BS in a 17 year old boy with Asperger's syndrome.**

History

SB was born uneventfully by LSCS at 36 weeks following a pregnancy complicated by placenta praevia and intermittent vaginal bleeding. He was diagnosed with sagittal suture stenosis and scaphocephaly at age 3/12. Quaternary follow up at 3 years found his development normal; however SB's mother recalls that as a pre-schooler he "couldn't remember or retain information about shapes. At 5 years paediatric examination identified severe generalised hypotonia and noted that SB had issues with social-communication with peers. Tertiary neurology and genetics referrals identified no cause for SB's hypotonia; investigations included MRI of brain (and later spine) and peripheral nerve conduction tests. Later quaternary referral excluded a primary muscle disorder. At 11 years Asperger's syndrome (ICD10 criteria) was diagnosed following multidisciplinary assessment. Tertiary neurological confirmation of BS was made when SB was aged 13 years and recommendations were made to support him as a child with severe visual perceptuomotor disability. At 14, SB developed severe anxiety and depression. Psychiatric referral led to treatment with first sertraline, then fluoxetine, which continues.

Examination Methodology

SB underwent ophthalmic and standardised visual perceptual assessments, standardised motor skills assessment, and neurological examination that included examination for OA.

Ophthalmic /orthoptic examination

Visual acuity (Keeler crowded logMAR); colour vision (Ishihara); stereoacuity (Frisby stereotest); eye movement assessment and cover test for strabismus; visual field assessment (confrontation testing and static field perimetric assessment); retinoscopic examination.

Standardised assessments of vision perception

1. The Beery VMI Developmental Test of Visual Perception[7].
2. The CVI Inventory[8] (Fig.3), a standardised parental interview that detects dorsal stream visual dysfunction (DSD) and ventral stream dysfunction (VSD).

Standardised assessment of hand motor coordination skills

The Beery VMI Developmental Test of Motor Coordination[7].

Neurological examination

Standard neurological examination included testing for cerebellar and anterior parietal function.

Examination for optic ataxia:

1. Criterion for diagnosis of optic ataxia and rationale

- Normative data[9,11] on the development of hand grasp-accuracy in childhood gives 2½-3 years as the lower limit of skill acquisition; the upper being 4 years[10-12].
- We have concluded that the adult-derived criterion of **impairment of terminal grip size to target**[13-17] can (in the absence of other visual, sensory, or neuromuscular impairment), reliably diagnose optic ataxia.

2. Standard procedure for examination for optic ataxia

- This has been adapted from methodology reported in the adult[18] and paediatric literature[11] for use in the paediatric age range 4-16 years.
- Immediate grasp in central vision is tested for a standard array of novel targets (see Fig.4) and filmed using video (25fps).
- Peripheral grasp is assessed as outlined in Fig.4.
- Typically developing controls (age range 8-16 years) without motor or visual perceptual impairment do not show impairment of grasp for the same grasp conditions.[19]

Video analysis

Video was examined for evidence of OA using slow-motion capture and/or still-frame analysis of filmed sequences, after Jeannerod[18].

Cognitive assessment

Weschler –III results for SB, aged 11y	Result	Percentile
Verbal IQ	96	37
Performance IQ	64	1
Full Scale IQ	77	6

Weschler –III : Performance Subtest Results

Subtest	Score	Age Equivalent
Picture completion	1	<6y
Coding	5	8y-8y3m
Picture arrangement	3	6y4m-6y7m
Block design	4	<6y
Object assembly	9	11y4m-11y7m

Weschler –III : Verbal Subtest Results

Subtest	Score	Age Equivalent
Information	9	10y-10y3m
Similarities	8	9y4m-9y7m
Arithmetic	17	>16y7m
Vocabulary	13	11y4m-11y7m
Comprehension	9	7y3m

Visual Behavioural Interview:
The CVI Inventory

51 questions, 5 forced choice responses:
never/rarely/sometimes/often/always
7 subsections for impairments in:
- visual field or field attention (lower/hemifield) [DSD}
- handling visual scene complexity [DSD}
- movement perception [DSD}
- visually guided movement [DSD}
- visual attention [DSD}
- coping with crowded environments [DSD}
- recognition and orientation [VSD}

Commonest patterns in CVI: DSD>DSD+VSD>VSD

Fig.3

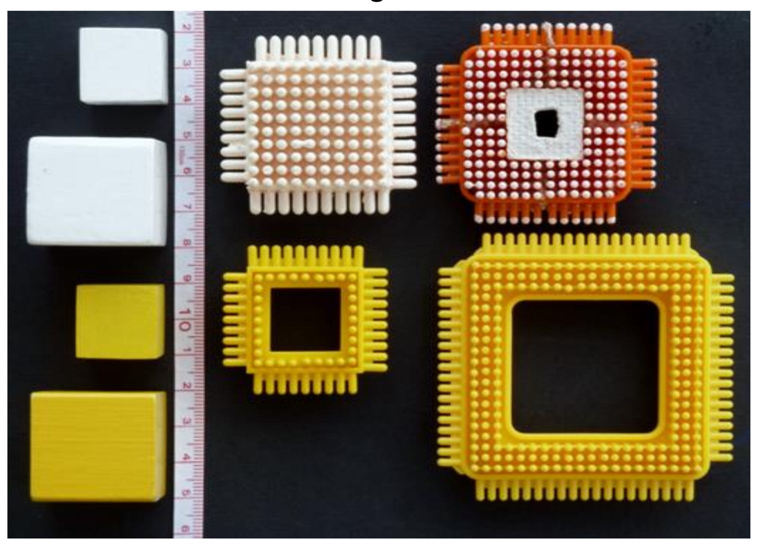

Fig.4: Immediate grasp of an object requires the simultaneous visual perception of its component features such as colour, texture, orientation[20,21]. Objects shown are presented in a standard order, top to bottom, left to right.

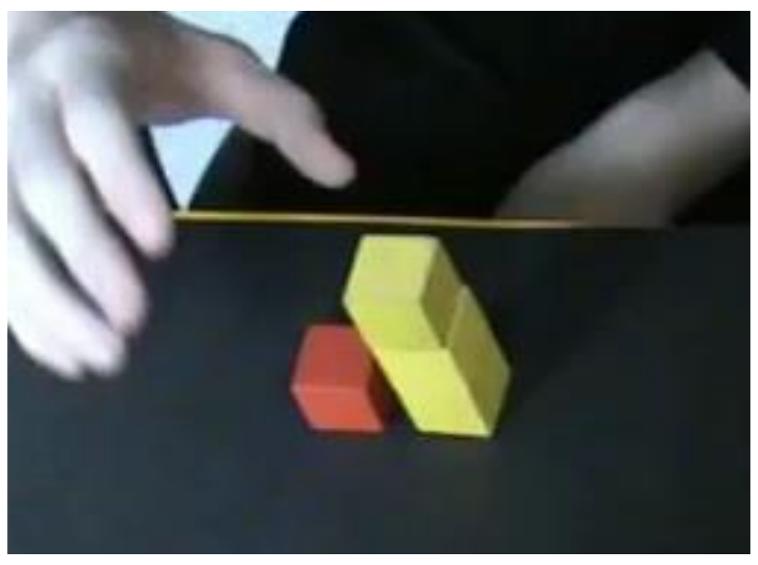

Fig.5: Obstacle avoidance task (target approx 15cm from body). The task is to sequentially transfer each cube of a 3cm cube tower to a new tower constructed in the opposite hemispace, then transfer the cubes back again. The instruction is to "keep looking at the central cube if you can". Grasp in peripheral vision requires confirmation of gaze fixation on the central item.

Results

Ophthalmology Results

Standardised visual perceptual assessment (Beery VMI) at 6 years 3 months rated perception for SB on the 97th percentile. Functional vision profile at 11 years (Table 1) indicated issues with peripheral vision. Initial ocular examination at 12 years was unremarkable and automated visual field assessment normal. SB's central "high contrast" visual acuity at presentation was, and continues to be 6/6.

At 15 years follow up ocular examination was normal but deteriorating fields were noted on perimetry (Fig 6). At 17 years pallor of the left optic disc was noted in association with progressive field impairment on perimetry (Fig 7). Electro-retinography has been requested.

CVI Inventory (Table I) At 11 years SB's parent reported impaired function in all four areas of dorsal stream dysfunction (movement perception, simultaneous perception, visual attention and visually guided movement); DSD was therefore rated as severe. Difficulty recognising faces, objects and familiar environments was also reported for SB. We classed this as a "dorsal stream plus" (DSD+)[22] dysfunction.

Parental Reports of DSD/ DSD+ Impairments on CVI Inventory @ 11y

	DSD Category		Significant Impairment
DSD	Visual attention	Both hemifields	+
	Handling the complexity of the visual scene		+
	Perception of movement		+
	Visually guided movement		+
	Face expression processing		+
DSD+	Recognition and navigation (familiar environments)		+

Table 1

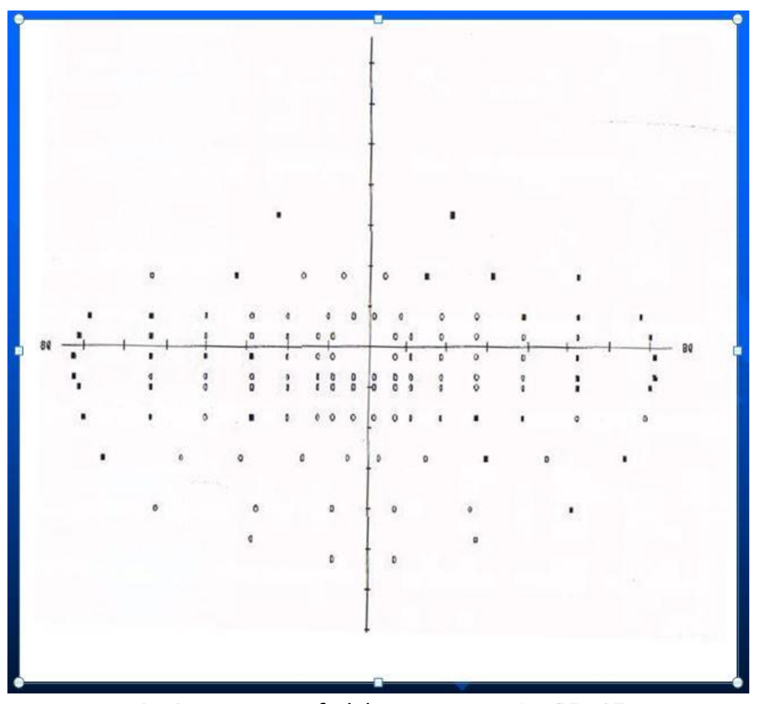

Fig.6: Concentric field restriction in SB, 15 yrs (binocular). Targets seen 84/120; not seen 36/120.

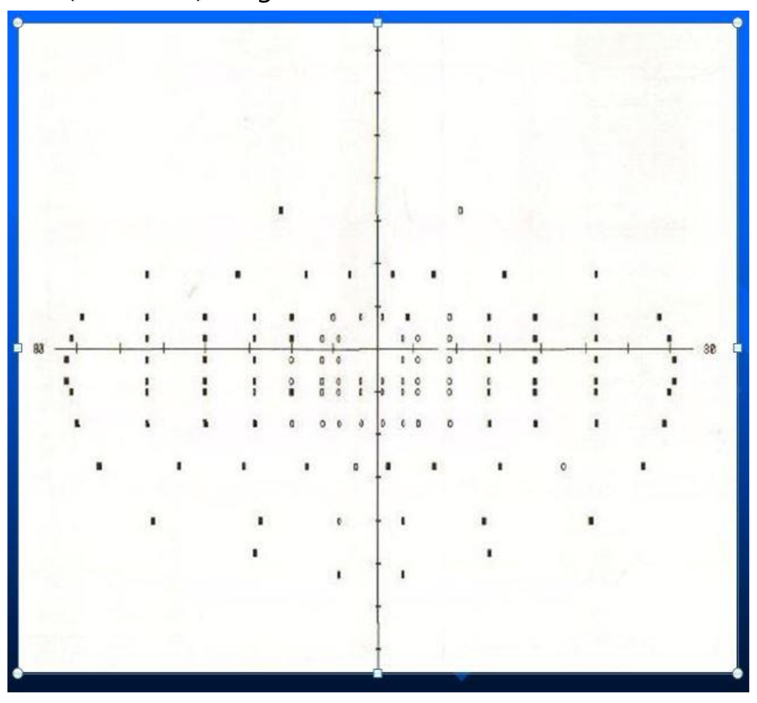

Fig.7: Progressive field limitation in SB, 17 yrs. Targets seen 44/120; not seen 76/120.

Motor coordination assessment and neurological examination

At 6years 3months occupational therapy assessment rated hand motor skills (using the Beery VMI) on the 0.8[th] percentile. When SB was aged 13 visual perceptuo-motor examination identified simultanagnosia on visual behavioural interview. Bilateral central OA of the upper (Fig 8) and lower limbs was diagnosed by slow motion video capture at 13 years. Repeat examination when SB was aged 16 years and nine months found him unable to visually identify shapes in a standard wooden formboard (achievable visually by 8 years) without using touch. Visual shape identification of the same items had been effortless at 11 years. OA was more severe (Fig 9) with increased duration of grasp and increased tactile exploration for objects (shown below for a 3cm yellow cube).

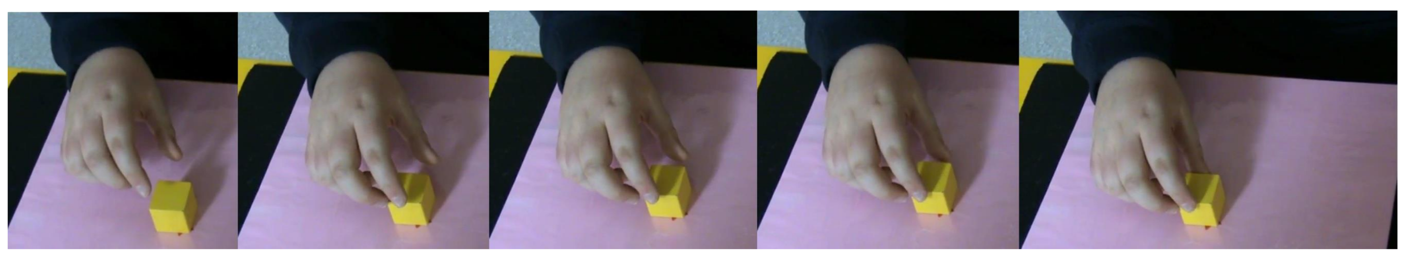

Fig.8: RH Grasp sequence for a 3cm yellow cube at 13y. Sequence duration: 1.33s; minimal tactile exploration prior to final grasp.

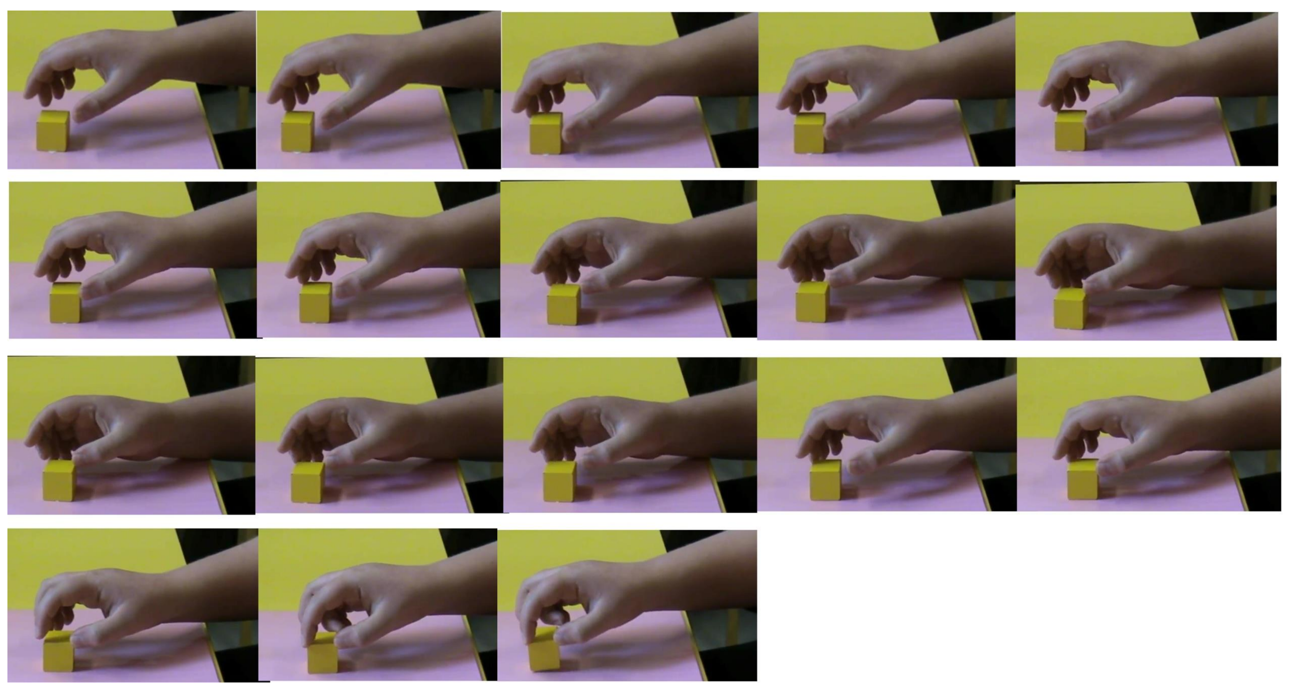

Fig.9: RH Grasp sequence for a 3cm yellow cube at 16y 9m. Sequence duration: 7.820s; extensive tactile exploration prior to final grasp.

Discussion

There is extensive evidence for a "dorsal stream vulnerability" in the developmental disorders[23]. In autism two recent studies add to this body of evidence with report of reduced functional visual fields[24] and reduced peripheral field sensitivity[25] in ASD.
We know of a single verified case report of an adult with abnormal ERG findings where the possibility of a causal link to SSRI treatment has been considered[26]. Nothing is known about the effects of SSRI medication on visual perception although associations with motor disorder have been reported[27,28].

Children with developmental disorders , including those with autism, and coexisting motor impairments may have visual perceptual as well as motor impairment. This is the first case we have seen with progressive deterioration in motor and visual perceptual function and the aetiology of this deterioraton remains uncertain. A causal link to SSRI treatment cannot presently be excluded.

References

Available on request

Advisors to current project looking at CVI in autism and contributors to CVI service

Professor Gordon N Dutton
Dr Paul Eunson
Dr Gwen Baxter, Research and Development, NHS D&G
Debbie Cockburn
Catriona Macintyre Beon
Colleagues in Ophthalmology and Orthoptics, and Paediatrics, NHS D&G
Colleagues in Speech and Language Therapy, Occupational Therapy and Physiotherapy
Colleagues in Educational Psychology and VI teaching service, Dumfries and Galloway Council
Most of all, thanks to the children, and to their parents
