# Supplementary material for: Emulation of the subjective experience of visual dorsal stream dysfunction: a description of three in depth case studies
Source: Front Hum Neurosci. 2025 Jan 6;18:1496811. doi: 10.3389/fnhum.2024.1496811 (PMC11743676; doi:10.3389/fnhum.2024.1496811)
Supplement: Supplementary file 3 [file Data_Sheet_3.docx]

Appendix 3

Verbatim responses to videos.

Case 1 (2019)

**Shop (1)**

Wow that’s fast

I’ve automatically drawn my face away, straight away.

So I can see one teddy bear

There’s a, it’s gone

I didn’t get to see what was on the teddy bears. I’m trying to see what’s on the teddy bears, um, jersey and now I’ve completely lost track of where I’m up to because I wanted to see what was on the jersey that I didn’t see, so now I’m not even focusing on anything.

Ok, so the University of St Andrews t-shirt, a white one and I don’t know what size that is, I can’t see what size.

There something on the ground there, underneath the things, little sign

Back to the, err, I’m not really focusing on anything to be honest.

Umm, damn teddy bears.

I can’t see anything on that, like, those little things on that stand, there all too little I can’t distinguish them, it’s too fast.

There’s a sign at the front of the counter, the desk, the counter, I can see Christmas

Case 2

**Beach (2)**

Sand, rocks, trees, tail, two people kicking a ball, dog in the center

**Driving (1)**

Driving through a town, a few road signs, oncoming bus, oncoming cars, trees, cars, parked car, white building, peculiar shape, roads, traffic

**Shop (1)**

Bears, clothes, Uni St Andrews shirts, focusing on shirts, picking up one, moving

**Beach (1)**

Rocks, same people, dog seen earlier, wheelchair, someone in it, another dog, rocks, mountain

**Driving (2)**

Town, lot of things going on, buildings, following a large object, probably bus, few people on sidewalks, spire,

traffic lights, cars

**Shop (2)**

Clothing, University of St Andrews, take something, letters on clothes, someone briefly appeared

**Shop (1) with Simultanagnosia Simulation Filter**

Kids section, bears again, clothes again, I can see more I can see everything, clothes, moving through shop, cash register

Case 3

**Beach (2)**

Sand, a dog, a setter or a spaniel I think, it’s playing with a green ball, there’s two people with the dog

**Shop (1)**

Teddy bears, some clothing, infant stuff, more clothing, lots of displays, tills, sales assistant

**Driving (1)**

Traffic lights, traffic cones, double decker bus, some cars, a dog, some trees, a van, bus stop, some more cars, a pink van, more traffic, some houses, traffic lights

**Beach (1)**

Some rocks, sand, a black labrador, wheelchair, person, red setter, some trees, the sea

**Driving (2)**

Double decker bus, blue car, some buildings, traffic, a bus

**Shop (2)**

Some t shirts, red cap, blue cap, some tops, a pink top

**Shop (1) with Simultanagnosia Simulation Filter**

Teddy bear, some baby clothes, pink jumper, some displays, a till

Case 1 (2024)

**Shop (1)**

Oh, it’s so, it’s too fast, black teddy bear, no a teddy wearing a black t-shirt, teddy bears with red and blue, sports jacket, baby clothes, St Andrews jerseys, green ones, a white St Andrews t-shirt, coat hangers with like red, blue and maybe gr…I don’t know what the last color was, continuing to walk through the shop, teddy bears are on the left, there’s like a sign or a plant or something underneath the table, jerseys on the right, it’s too fast, lost my words, wooden floor, shelving, like a desk, like a card machine and bags, someone behind the counter.

**Beach (1)**

Rocks, there is a, there was a mountain or an island in the distance, wheelchair, person wearing a blue hat, a black labrador with a, maybe a, doesn’t really matter what color the collar is. Can’t see. Trees, sand, another mountain or island in the distance, a bird I think just flew across,

**Driving (2)**

A double decker bus in front and then glare on the window, arrow blue arrow sign, blue car, another blue arrow sign, white van coming towards us, people walking down the street, a car moving infant of us, another bus going in the other direction, a steeple, and a maybe, I don’t know whatever you call it, a clock tower, another steeple, a traffic light, a red van going in front.

**Driving (1)**

Traffic light turning green, a tree, lamppost, a, maybe a bus in the distance, another double bus, cars parked, a person walking a dog I think, trees, another person walking, maybe, like some road signs, I don’t know, they’re different to our road signs, white car pulling out in front, there’s a big white building in the distance, just trees on the side, bit like a bus-stop, a big road-sign with the roundabout, car coming in front of us at the roundabout, a red car, white van, there’s cars on the other side, a blue car parked, coming up to another traffic light which is green, a lamppost.

**Beach (2)**

Footprints in the sand, rocks, sky, trees, there’s that island in the distance with the hill, big hill, and lots of rocks, birds flying around, there’s the water, obviously the tides going out, oh and there’s someone playing football, green football, another person’s turned up, and the black dog

**Shop (2)**

Lots of t-shirts, a beanie I think I saw a grey beanie, a hat, an extra small t-shirt long sleeved top, and then a small one, and then another small one and then a medium one on coat hangers, there’s a person coming towards you.

**Shop (1) with Simultanagnosia Simulation Filter**

Umm, it’s too fast, teddy bears, a blue teddy bear a red teddy bear a blue teddy bear a red teddy bear, more red and blue teddy bears, lots of teddy bears, a blue like jacket and a red jacket, a, looks like baby clothes, um, yer, no idea, just clothes and a rack, must be a t-shirt, university, there’s red labels but I can’t see what they say, I can’t see what size it is, that’s the floor, there’s some shelving, no, there’s I think it’s a sign, umm it’s going too fast, are they snow globes?, like t-shirts on a bottom shelf, is that like a box? No, it’s a counter.

Comparison

**Shop (1)**

Some hoodies, some teddy bears with some university logos on, more teddy bears with a hat, red covers, red hoodies, I’m now Looking at baby grows on hangers with a little tiny teddy bear, and there’s some sweatshirts, green, grey, blue, mauve, maroon, a University of St Andrews or St Andrews white, hangers size …size age seven to eight I think, more grey ones, a hanger there, some blue ones, that’s seven to eight was picked up and chosen there. I’m going along a wooden floor, I’m going past the teddy bears again, some snow globes of St Andrews, some St Andrews sweatshirts and t-shirts there, I can see more hoodies, I can see some things like a bobble hat, more coat hangers or bags, jewelry, I can see some more St Andrews paraphernalia, I can see a counter here, I can see a Christmas sale, there’s a man, there’s a man looking down at a giraffe

**Beach (1)**

I’m at the beach, I can see rocks, I can see a ship or something way in the distance there, I can see a dog, a buggy, a person in the buggy, I can see a person sitting on the sand with a blue hat, I can see a green ball, I can see a flat moving, oh I can see another doggy bouncing around wagging its tail, I can see seaweed, more rocks, I can see some birds floating into the sky here, I can see what looks like North Berwick Law, think it could be, yep

**Driving (2)**

I’m on a street, I can see a bus, the 23 bus that goes to trinity, I’m following a number 11 bus that I think goes to Hyvots Bank whatever that is, tempo perso *[shop name] I* know where I am in Morningside, can see a car parked on the left hand side, flashing indicators, a van I can see some outdoor bins there, the car’s just moved off infant of me SH61 WDV, I can see some church spires at the top, number 45 bus, beginning to overtake and go in the middle of the road of that bus there, there’s some shops, ‘everyone’s designs is on the left hand side, there’s some red traffic lights, I’m stopping there, I can see a big ‘keep clear’ sign, I’m looking at a big church spire on my left and on the right.

**Driving (1)**

I’m now going through three sets of traffic lights, they’re all on green, I can see a ‘go this way’ sign, I’m heading towards, straight down the road, some roadworks there, a bus is coming towards me, a grey car is parked on the right hand side, I can see a bus stop with a blue bin or a grey bin, there’s a blue bin there, again ‘keep left’ sign, a man with a dog there, some white cars parked on the left hand side, there’s a man with a shopping bag or woman there, blue van has just gone past, British Gas or something like that, I can see a pedestrian crossing, a grey car a white van on the right hand side, there’s a park to the left hand side of me, a bus stop to the right hand side, another grey car, a white car that’s just turned left, bus has just pulled out in front, I can see a building on the right a bus stop on the left, I ‘m coming up to a roundabout, there’s a mast for mobile phones just past, city center turn right, we’re going right, we’re following the sign to the city center, there’s more grey cars, I’m at a roundabout a red mini, a white car, I’m turning right, a Porsche’s just stopped, we’re going up to the city center, I can see some traffic lights, some cars, a white BMW’s parked on the left hand side, some trees now I can see some houses and I’m going up a hill

**Beach (2)**

So I’m at the beach, I can see some trees, North Berwick Law, some rocks, possibly some seaweed, there’s some seagrass at the edge, a birds just flown over a dog’s just come into view, like something like an Irish Setter, there’s a person with a blue wooly hat and a hoody playing with a green football, there’s a black dog playing football as well, and the dog has taken the ball, seaweed on the beach

**Shop (2)**

I’m in a shop I’m looking at, I guess a university of St Andrew’s shop, I’ve got some t-shirts, some hats to the left of me, some things on a hanger, it’s a white hoody jumpery thing, extra small, small medium and something at the back there as well, they’re on hangers, there’s one two three four, somebody’s just picked the medium size I guess I think, baseball, baseball hat again, I think St Andrews, there’s a man, a man coming up to me, he’s disappeared

**Shop (1) with Simultanagnosia Simulation Filter**

I’m in a shop and there’s some teddy bears, blue teddy bears, red teddy bears, again red teddy bears with the hoodies on, I’m now looking at some pink baby clothes, there’s a wee teddy bear there, a grey hoodie a green hoodie a red hoodie, a white university of St Andrews on it, coat hangers, small, grey hoodies, age thing, coat hanger, someone’s picked something, seven to eight I assume past the pink hoodies and the baby grows, going towards a sign, that says, I don’t know what that says, university of St Andrews but backwards I think, soap or something like that, snow globes, I’m looking around I can’t really see anything that’s going on, I can see some jewelry, more hoodies with St Andrews, I can see some soap maybe, I can see a sign that says something, oh Christmas sale 1maybe I can see Christmas sale I can see a card machine
